# Metabolic engineering of *Pseudomonas putida* for production of vanillylamine from lignin‐derived substrates

**DOI:** 10.1111/1751-7915.13764

**Published:** 2021-02-03

**Authors:** João Heitor Colombelli Manfrão‐Netto, Fredrik Lund, Nina Muratovska, Elin M. Larsson, Nádia Skorupa Parachin, Magnus Carlquist

**Affiliations:** ^1^ Grupo Engenharia de Biocatalisadores Instituto de Ciências Biológicas Universidade de Brasília Brasília Brazil; ^2^ Division of Applied Microbiology Department of Chemistry Faculty of Engineering Lund University PO Box 124 Lund 221 00 Sweden; ^3^ Department of Bioengineering California Institute of Technology 1200 East California Blvd Pasadena CA 91125 USA; ^4^ Present address: Applied Microbiology Lund University Kemicentrum, Naturvetarvägen 14 Lund 22100 Sweden; ^5^ Present address: Ginkgo Bioworks 27 Drydock Ave Boston MA 02210 USA

## Abstract

Whole‐cell bioconversion of technical lignins using *Pseudomonas putida strains* overexpressing amine transaminases (ATAs) has the potential to become an eco‐efficient route to produce phenolic amines. Here, a novel cell growth‐based screening method to evaluate the *in vivo* activity of recombinant ATAs towards vanillylamine in *P. putida* KT2440 was developed. It allowed the identification of the native enzyme Pp‐SpuC‐II and ATA from *Chromobacterium violaceum* (*Cv‐ATA*) as highly active towards vanillylamine *in vivo*. Overexpression of *Pp‐SpuC‐II* and *Cv‐ATA* in the strain GN442ΔPP_2426, previously engineered for reduced vanillin assimilation, resulted in 94‐ and 92‐fold increased specific transaminase activity, respectively. Whole‐cell bioconversion of vanillin yielded 0.70 ± 0.20 mM and 0.92 ± 0.30 mM vanillylamine, for *Pp‐SpuC‐II* and *Cv‐ATA*, respectively. Still, amine production was limited by a substantial re‐assimilation of the product and formation of the by‐products vanillic acid and vanillyl alcohol. Concomitant overexpression of *Cv‐ATA* and alanine dehydrogenase from *Bacillus subtilis* increased the production of vanillylamine with ammonium as the only nitrogen source and a reduction in the amount of amine product re‐assimilation. Identification and deletion of additional native genes encoding oxidoreductases acting on vanillin are crucial engineering targets for further improvement.

## Graphical abstract

In this study, *Pseudomonas putida* was engineered for whole‐cell conversion of ferulic acid and vanillin to vanillylamine with ammonium as nitrogen source. Furthermore, a novel growth‐based screening method to select for recombinant vanillin transaminases was developed and used to identify SpuC‐II and Cv‐ATA as suitable enzymes for in vivo biocatalysis with *P. putida*.

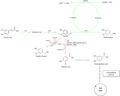

## Introduction

Amines are essential chemical building blocks in the chemical industry used to produce various pharmaceuticals, agrochemicals, cleaning agents, personal care products and polymers (Froidevaux *et al*., 2016; Kelly *et al*., 2018). Most amines produced industrially are derived from platform chemicals of fossil origin. Nevertheless, there is a growing interest in developing bio‐based production processes from non‐food renewable resources following the green chemistry principles (Masuo *et al*., 2016; Zhou *et al*., 2018; Blondiaux *et al*., 2019). In recent years, technical lignins, such as Kraft lignin, lignosulfonates, soda lignin and organosolv lignin, have been recognized as an abundant potential source of platform chemicals through bio‐refining (see previous reviews (Abdelaziz *et al*., 2016; Wang *et al*., 2019)). Microbial bioconversion of depolymerized technical lignin (DTL) to various products, such as polyhydroxyalkanoate (Salvachúa *et al*., 2020), muconic acid (Kohlstedt *et al*., 2018; van Duuren *et al*., 2020), lipids (Bhatia *et al*., 2019) and others (reviewed by Becker and Wittmann (2019)) have been developed. Bioconversion of DTL to amines has, however, been studied little.

Bio‐catalytic amine production can be achieved using amine transaminases (ATAs), which is now the preferred method in the pharmaceutical industry (Bryan *et al*., 2018). ATAs are pyridoxal 5'‐phosphate (PLP)‐dependent enzymes (E.C. 2.6.1.x) that catalyse reversible transamination of carbonyl compounds through a ping‐pong bi‐bi mechanism (Patil *et al*., 2018). Unfavourable thermodynamic equilibrium towards the amine product and enzyme inhibition by (co‐) products often hinder reaction efficiency. Thus, strategies for surpassing this typically involves using an excess amount of amine donors (such as l‐alanine or isopropylamine) and/or coupled reactions to remove the co‐product or to recycle the amine donor (Wu *et al*., 2016; Wu *et al*., 2017a; Zhou *et al*., 2018). Whole‐cell transamination (Weber *et al*., 2014a; Weber *et al*., 2017; Patil *et al*., 2019; Molnár *et al*., 2019) offers several advantages over *in vitro* because it supplies PLP and amine donor, recycles cofactors, removes co‐product, as well as simplifies upstream preparation of the biocatalyst, which altogether result in significant process improvement (Tufvesson, Lima‐Ramos, Jensen, *et al*., 2011; Tufvesson, Lima‐Ramos, Nordblad, *et al*., 2011). On the other hand, whole‐cell systems are often sensitive to adverse process conditions, such as high titres of inhibitory substrates and products. They may harbour native activities resulting in by‐product formation (Straathof *et al*., 2019). In the case of ATA‐based whole‐cell production of amines, identification and knock‐out of native enzymes that acts on the carbonyl substrate are typical engineering targets.

**Fig. 1:**
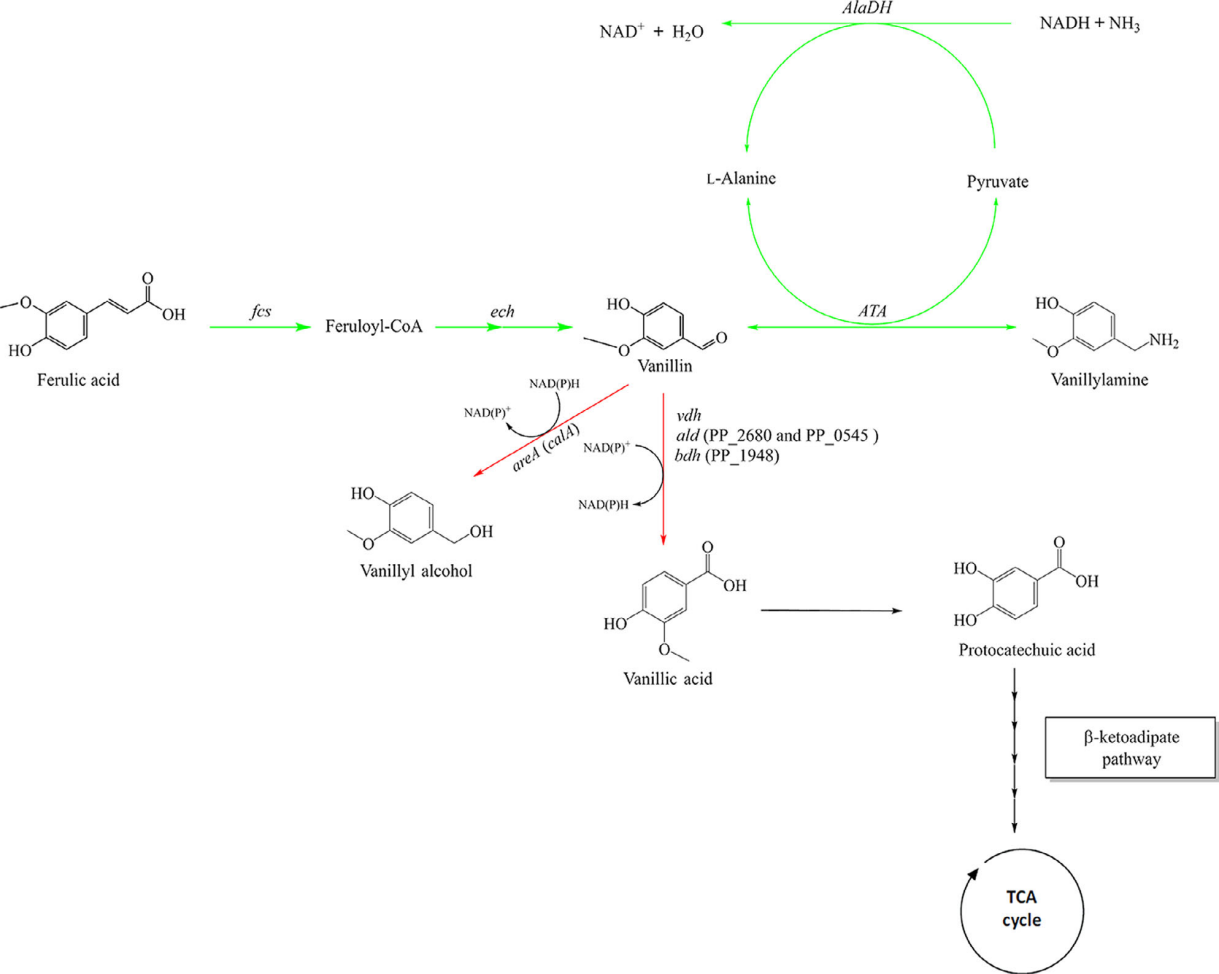
Biochemical route for conversion of ferulic acid to vanillylamine, and major by‐products vanillyl alcohol and vanillic acid. The first step is the synthesis of vanillin from ferulic acid by the two enzymes Feruloyl‐CoA‐synthetase and Enoyl‐CoA‐hydratase/aldolase encoded by the genes *fcs* and *ech*, respectively. The reversible transamination of vanillin is accomplished by an amine transaminase (ATA). The amino group is transferred from the amino donor (l‐alanine) to vanillin with the formation of the corresponding amine and release of a co‐product (pyruvate). Alanine dehydrogenase (AlaDH) recycles l‐alanine from pyruvate consuming NADH and NH_3_. Conversion of vanillin to vanillyl alcohol, and to vanillic acid is prevented by the deletion of vanillin dehydrogenase (*vdh*), and the aldehyde dehydrogenases (PP_2680 and PP_0545) and *bdh* (PP_1948; Graf and Altenbuchner, 2014), as well as *areA* (*calA*) encoding a vanillin reductase (García‐Hidalgo *et al*., 2020). Black arrows: Not modified; Green arrows: overexpressed genes; Red arrows: deleted genes.

*Pseudomonas putida* is a gram‐negative bacterium with high tolerance to aromatic compounds, and it carries several well‐characterized funnelling pathways for the assimilation of various aromatics (Brink *et al*., 2019; Xu *et al*., 2019). Furthermore, it is a somewhat industrially domesticated bacterium with a wide range of genetic tools for metabolic engineering (Aparicio *et al*., 2020; Wirth *et al*., 2020; Batianis *et al*., 2020), as well as suitable strategies for bioprocess scale‐up (Nikel and Lorenzo, 2018). Altogether this has resulted in *P. putida* being one of the most preferred platform hosts for the valourization of DTL (Becker and Wittmann, 2019). Metabolically engineered *P*. *putida* has previously been demonstrated to be efficient in converting DTL to various carboxylic acids, for example muconic acid, pyruvic acid, lactic acid and 2,5‐furandicarboxylic acid (Johnson *et al*., 2019; Weimer *et al*., 2020). To the best of our knowledge, the use of *P. putida* for bioconversion of DTL to phenolic amines has, however, not been studied previously. Herein, *P. putida* was engineered and evaluated for whole‐cell bioconversion of vanillin and ferulic acid to vanillylamine (VA; Fig. 1). The interest in VA comes from its use as intermediate in the synthesis of bioactive compounds, such as capsaicinoids (Sudhakar Johnson *et al*., 1996; Anderson *et al*., 2014; Arce‐Rodríguez and Ochoa‐Alejo, 2019), and as a precursor for the production of polyepoxides (Fache *et al*., 2015; Mogheiseh *et al*., 2020).

*Pseudomonas putida* KT2440 has several native enzymes that use vanillin as substrate, including vanillin dehydrogenase and several aldehyde reductases resulting in the formation of the by‐products vanillyl alcohol or vanillic acid (Simon *et al*., 2014), with the latter being further assimilated via protocatechuate and the β‐ketoadipate pathway (Fig. 1). A more suitable host strain than KT2440 for production of VA may therefore be GN442ΔPP_2426, which was previously engineered to produce vanillin from ferulic acid (Graf and Altenbuchner, 2014; García‐Hidalgo *et al*., 2020). In this strain, the native genes coding for vanillin dehydrogenase (*vdh*), two aldehyde dehydrogenases (*aldB‐II* (PP_2680) and *aldB‐I* (PP_0545)), a benzaldehyde dehydrogenase (PP_1948) and the coniferyl alcohol dehydrogenase *calA* (PP_2426) were previously deleted (García‐Hidalgo *et al*., 2020; Fig. 1). Also, genes coding for feruloyl‐CoA synthetase (*fcs*) and enoyl‐CoA hydratase/aldolase (*ech*) are also overexpressed, resulting in the improved conversion of ferulic acid to vanillin (Graf and Altenbuchner, 2014). Under non‐growing conditions, ferulic acid was converted to vanillin with 82% yield (mol/mol) using this strain (García‐Hidalgo *et al*., 2020).

In the present work, three native and two non‐native ATAs were evaluated for *in vivo* activity towards VA in *P. putida*. For this purpose, a novel growth‐based screening method suitable for high‐throughput evaluation of candidate genes was developed. Identified genes encoding the most active enzymes, putrescine transaminase Pp‐SpuC‐II (Galman *et al*., 2018) and the well‐known ATA from *Chromobacterium violaceum* (Cv‐ATA; Kaulmann *et al*., 2007) were overexpressed in the strain GN442ΔPP_2426 (García‐Hidalgo *et al*., 2020). Furthermore, the effect of coexpressing Alanine dehydrogenase (AlaDH) from *Bacillus subtilis* for the regeneration of l‐alanine was evaluated. Our findings provide a proof‐of‐principle that *P*. *putida* with engineered ATA activity in combination with reduced vanillin assimilation indeed can be used for VA production using ammonium as a nitrogen source and without supplementation of l‐alanine in the reaction broth. Still, adverse oxidoreductase background activities converting vanillin to vanillic acid and vanillyl alcohol remain key bioengineering targets to reach increased titre and yield.

## Results and discussion

### Development of a growth‐based method to measure transaminase activity towards vanillylamine in *P. putida* KT2440 and identification of ATAs

**Fig. 2:**
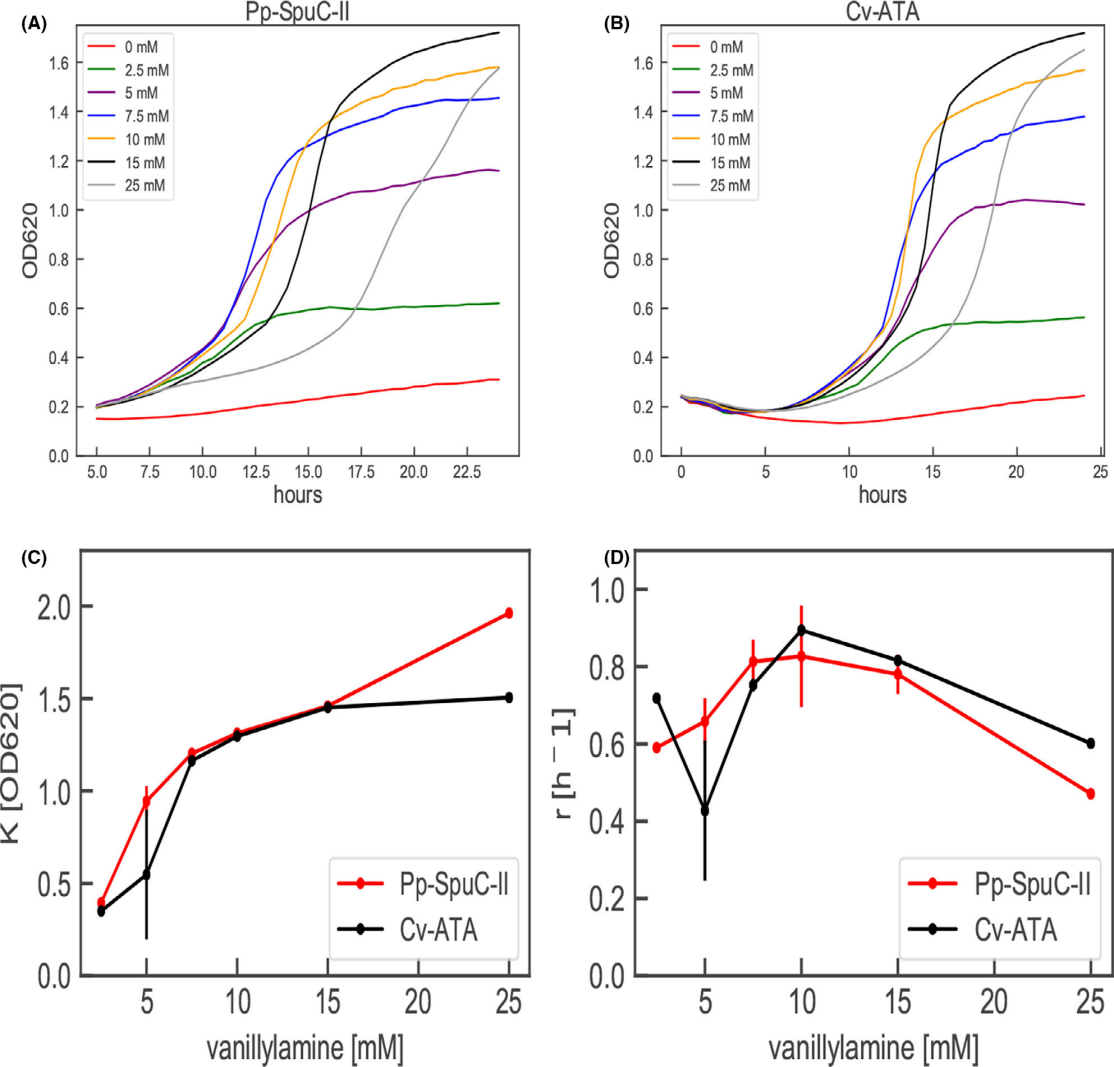
Growth of *P. putida* KT2440 overexpressing (A) Pp‐SpuC‐II (TMB‐NM011) or (B) Cv‐ATA (TMB‐JH002) in M9 medium supplemented with different concentrations of vanillylamine (0–25 mM) as sole carbon source. Growth curves are representative figures from one of the two biological replicates. After fitting the data in (A–B) to a logistic growth curve (described in Experimental Procedures) the estimated Carrying capacity (K; Panel C) and the maximal growth rates (r; Panel D) for each concentration are plotted against vanillylamine concentration for both Pp‐SpuC‐II (red line) and Cv‐ATA (black line).

*Pseudomonas* spp. are known to metabolize biogenic amines (see review by Luengo and Olivera (2020)). Also, several natural isolates of *P. putida* were previously found to grow using VA as the sole carbon source (Flagan and Leadbetter, 2006), indicating that endogenous transaminases can convert VA to vanillin, which is assimilated via the *β*‐ketoadipate pathway. *In vivo* evaluation of ATAs by growth on specific amines as a carbon source is a powerful high‐throughput screening tool to facilitate whole‐cell biocatalyst development. Here, a *P. putida* KT2440 whole‐cell assay was assessed by determining cell growth profiles in liquid M9 medium supplemented with glucose, vanillin or VA as the sole carbon source. No growth was observed for *P. putida* KT2440 (wild‐type) during 48 h of cultivation with VA as the only carbon source, while significant growth with vanillin (final OD 0.8 ± 0.0) was evident (Fig. S1). This indicates that (i) the endogenous ATAs lack activity towards VA or (ii) that the endogenous ATAs are expressed at an insufficient level to sustain cell growth under the applied conditions. To investigate which of the two alternatives was the case, we searched for putative native transaminases in *P. putida* KT2440 (taxid: 160488) by using BLASTp analysis (Dalal and Atri, 2014) with two ATAs as query sequences; Cv‐ATA from *Chromobacterium violaceum* (Kaulmann *et al*., 2007; Du *et al*., 2014) and Pp‐SpuC from *P. putida* (taxid: 303; Galman *et al*., 2017; Galman *et al*., 2018). Cv‐ATA (PDB id: 4BA5) was used as a query sequence since it has previously been found to catalyse the desired reaction (Kaulmann *et al*., 2007), and the putrescine transaminase Pp‐SpuC (PDB id: 6HX9) was chosen because it has been shown to recognize a broad spectrum of aromatic compounds as substrate (Galman *et al*., 2017; Galman *et al*., 2018). The top three ATAs displaying the lowest e‐value and highest sequence homology to both Pp‐SpuC and Cv‐ATA were chosen for further investigations and denoted here as Pp‐SpuC‐I (gene PP_2180), Pp‐SpuC‐II (gene PP_5182) and Pp‐ATA (gene PP_2588), respectively (Table S1). Genes encoding Pp‐SpuC‐II, Pp‐SpuC‐I and Pp‐ATA were cloned individually in the expression vector pSEVA424 carrying the IPTG‐inducible *lacI^q^/Ptrc* system. Plasmids were then transformed in the strain KT2440 to generate strains TMB‐NM011, TMB‐NM012 and TMB‐NM013, respectively (Tables S2 and S3). Also, the genes encoding Cc‐ATA (Weber *et al*., 2014b) and Cv‐ATA, both previously shown to accept vanillin as substrate, were cloned and transformed into KT2440 generating the strains TMB‐JH001 and TMB‐JH002, respectively (Tables S2 and S3). Constructed strains were evaluated for their ability to proliferate in liquid M9 medium supplemented with 5‐25 mM VA as the sole carbon source (Fig. 2).

The strains TMB‐NM011 and TMB‐JH002, overexpressing *Pp‐SpuC‐II* and *Cv‐ATA*, respectively, displayed significant growth, demonstrating that they carry the desired activity towards VA. However, no substantial growth was seen for the wild‐type or strains overexpressing *Cc‐ATA* (data not shown), *Pp‐SpuC‐I* and *Pp‐ATA* (Fig. S2). For the plant transaminase Cc‐ATA, previously found to be active when expressed in *E. coli* (Weber *et al*., 2014), this may be due to low expression level or protein misfolding in *P. putida* under the applied conditions. Furthermore, no negative impact on the growth with glucose in the medium was observed for the strains overexpressing the Cc‐ATA encoding gene (Figs S3 and S5), indicating that the lack of activity is not related to potential toxic effects of Cc‐ATA in *P. putida*.

Growth was similar for both strains overexpressing *Pp‐SpuC‐II* and *Cv‐ATA* on VA as limiting carbon source, and followed apparent Monod kinetics up to 15 mM with a growth rate plateau between 10 and 15 mM (Fig. 2C). Above this concentration, the cell growth rate was reduced (Fig. 2D); however, the carrying capacity was not affected (Fig. 2C), demonstrating no effect on final biomass yield (Fig. 2A and B). The high activity for Pp‐SpuC‐II was confirmed in subsequent whole‐cell bioconversion experiments where the conversion of VA in glucose‐grown cells was measured. The strain TMB‐NM011, overexpressing *Pp‐SpuC‐II*, displayed the highest specific transaminase activity (0.420 ± 0.03 mmol/h/OD compared to 0.020 ± 0.03 mmol/h/OD for the wild‐type), resulting in almost complete conversion of VA to vanillin within 6 hours (Fig. S3A). The specific transaminase activity for TMB‐NM012 and TMB‐NM013 was also higher than the wild‐type (0.052 ± 0.11 mmol/h/OD and 0.085 ± 0.00 mmol/h/OD, respectively) and reached complete conversion of 5 mM VA within 24 hours. For wild‐type KT2440, only 28% of the substrate was converted over the same period, demonstrating that native vanillin transaminases are weakly expressed in KT2440 under the studied conditions (Fig. S3D). Overall, the results suggest that all three native transaminases recognize VA as substrate in *P. putida* and Pp‐SpuC‐II showed the highest *in vivo* specific activity. However, the basal activity in the wild‐type strain is not enough to sustain growth on VA (Fig. S1).VA is not likely the natural substrate for the native ATAs presented here, although they have activity against VA in different levels. The Pp‐SpuC‐II, which is closely related to Pp‐SpuC from other *P. putida* strain (taxid: 303), is a putrescine transaminase that has preference for aliphatic diamines (Galman *et al*., 2017; Galman *et al*., 2018). Wu *et al*. (2017b) demonstrated the potential of Pp‐SpuC from *P. putida* NBRC 14164 for the kinetic resolution of several racemic amines and amino alcohols and corroborated its role as a promiscuous transaminase. In *P. aeruginosa* PAO1, the SpuC is the major putrescine transaminase and responsible for the conversion of putrescine to 4‐aminobutyraldehyde (Lu *et al*., 2002). Therefore, this indicates that Pp‐SpuC‐II (PP_5182) and/or Pp‐SpuC‐I (PP_2180) are promiscuous transaminases that may be related to putrescine metabolism in *P. putida* KT2440. Pp‐ATA (PP_2588) has been annotated as a Class III aminotransferase (Nelson *et al*., 2002), but has not been characterized previously. From sequence analysis in the conserved protein domain database (Lu *et al*., 2020), it is found to belong to the highly conserved aspartate aminotransferase family protein and it can be speculated to be a part of the amino acid metabolism in *P. putida* KT2440.

**Fig. 3:**
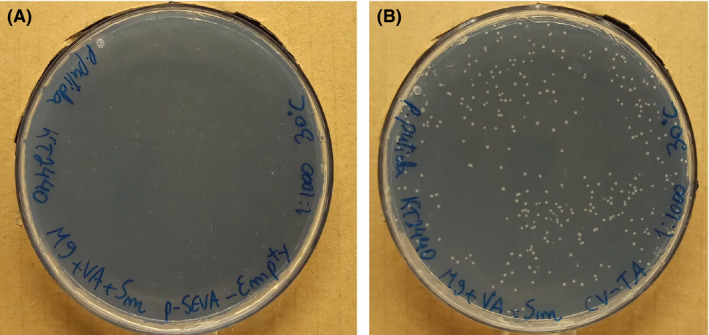
M9 plates after 3 days incubated at 30 °C with 5 mM of vanillylamine as the sole carbon source, streptomycin (100 µg ml^−1^) for plasmid selection and IPTG (1 mM) to induce gene expression. A: KT2440 cells with the empty pSEVA424 plasmid; B: TMB‐JH002 cells expressing *Cv‐ATA* gene.

Next, the possibility to identify vanillin transaminases by growing the recombinant cells on a solid medium as an integrated part of the transformation protocol was investigated. The constructed plasmid harbouring *Cv‐ATA* (pJH002) and the empty plasmid (pSEVA424) were again transformed into KT2440. The cells were poured directly onto M9 plates supplemented with 5 mM VA as the sole carbon source, streptomycin to select for the plasmid and IPTG to induce gene expression under the control of the *lacI^q^/Ptrc* system. Agar plates were incubated at 30 °C for 3 days and then evaluated for colonies' presence (Fig. 3).

There were no colonies on the plate with transformants bearing the empty plasmid (Fig. 3A), while small colonies were visible in the plate containing the cells expressing *Cv‐ATA* gene (Fig. 3B). The selection principle was thus found to be functional, that is only cells overexpressing the vanillin transaminase were able to sustain growth with VA as sole carbon source. However, addition of streptomycin to the medium was found to be necessary (Fig. S4), which may be explained by a correlation between the presence of the antibioticum and a sufficient plasmid copy number to ensure high enough expression level of the transaminase. A positive correlation between the concentration of antibioticum used for selection, the plasmid copy number and the expression level of a recombinant gene has been found previously (Begbie *et al*., 2005; Lian *et al*., 2016).

Nevertheless, the results demonstrate that it is possible to couple the growth‐based selection method directly to the transformation pipeline of *P. putida* KT2440. Hence, it can be used for high‐throughput screening of novel ATAs accepting VA as substrate or even developing evolutionary engineering studies of these enzymes allowing a better understanding of the structure–activity relationship. It may be possible to extrapolate the method for identifying ATAs able to convert other amines as long as the carbonyl product is metabolized by *P. putida* KT2440. Growth‐based screening methods for identifying genes coding for specific ATAs have to the best of our knowledge not been described previously. Other approaches using chemical analysis methods have been described (Baud *et al*., 2017; Pawar *et al*., 2018; Coscolín *et al*., 2019). However, they can be time‐consuming or require high‐cost reagents and/or equipment.

### Overexpression of transaminases in GN442ΔPP_2426 enables whole‐cell bioconversion of vanillin to VA

Genes encoding ATAs identified to carry the desired vanillin transaminase activity (*Cv‐ATA*, *Pp‐SpuC‐II*, *Pp‐SpuC‐I* and *Pp‐ATA*) and also *Cc‐ATA* were overexpressed in GN442ΔPP_2426. *In vivo* transaminase activity of constructed strains was assessed by whole‐cell bioconversion of VA to vanillin in M9 medium supplemented with glucose to verify if similar results as for KT2440 are obtained when the enzymes are produced by the strain GN442ΔPP_2426 (Fig. S5). Based on the *in vivo* assays, the initial conversion rates (first 6 h) were highest for strains overexpressing *Cv‐ATA* (0.276 ± 0.09 mmol/h/OD) and *Pp‐SpuC‐II* (0.281 ± 0.10 mmol/h/OD; Table 1; Fig. S5), which is in correlation with the observed growth on VA for the KT2440 strain background. These results indicate that both ATAs showed the highest specific transaminase activity against VA. No hyperexpression was observed in the SDS‐PAGE analysis performed in GN442ΔPP_2426 strains expressing different ATAs encoding genes (Fig. S8).

**Table 1:** Summary of results obtained from whole‐cell bioconversions performed using *P. putida* GN442ΔPP_2426 overexpressing different ATAs.

Strain	ATAs	*In vivo* specific transaminase activity in GN442ΔPP_2426^a^ (mmol h^−1^ OD^−1^)	Fold‐change in specific transaminase activity^b^	Maximum VA (mM)^c^/Time	VA Yield (mol mol^−1^; %)^d^
GN442ΔPP_2426 carrying the empty pSEVA424 plasmid	Negative control	0.003 ± 0.00^a^	1	0.010 ± 0.01^a^ 24 h	0.11 ± 0.16^a^
TMB‐NM014	Pp‐SpuC‐II	0.281 ± 0.10^b^	94	0.700 ± 0.20^b^, ^c^ 7 h	7.3 ± 2.6^a^, ^b^
TMB‐NM015	Pp‐SpuC‐I	0.032 ± 0.00^a^	11	0.180 ± 0.01^a^, ^b^ 24 h	1.9 ± 0.08^a^, ^b^
TMB‐NM016	Pp‐ATA	0.047 ± 0.01^a^	16	0.360 ± 0.01^a^, ^b^, ^c^ 6 h	3.6 ± 0.4^a^, ^b^
TMB‐JH004	Cv‐ATA	0.276 ± 0.09^b^	92	0.915 ± 0.30^c^ 2 h	9.9 ± 4.6^b^
TMB‐JH003	Cc‐ATA	0.002 ± 0.00^a^	ND	ND	ND

ND, Not determined.

Equal letters indicate no statistical difference between the samples (p > 0.05).

^a^ Values calculated based on the production of vanillin in the whole‐cell bioconversion of VA to vanillin using growing cells.

^b^ Values calculated in relation to the negative control

^c^ Values obtained in the whole‐cell bioconversion of vanillin to VA using resting cells. The time (h) indicates the period of bioconversion when the maximum VA titre was reached.

^d^ Calculated using the maximum VA obtained for each strain during the bioconversion of vanillin to VA (mol/mol) using resting cells.

Next, whole‐cell bioconversion of vanillin to VA was performed using resting cells in sodium phosphate buffer supplemented with l‐alanine as an amine donor (Fig. S6). All overexpression strains, except for *Cc‐ATA*, were capable of producing VA, although to a different degree, in correlation with specific transaminase activities measured in the reverse direction (Fig. S6F). The maximum VA concentration reached by TMB‐NM014 (Pp‐SpuC‐II) was 0.700 ± 0.20 mM, almost 4‐fold more than TMB‐NM015 (Pp‐SpuC‐I; 0.180 ± 0.01 mM) and almost twice the amount compared to TMB‐NM016 (Pp‐ATA; 0.360 ± 0.01 mM), while TMB‐JH004 (Cv‐ATA) produced the highest amount of VA 0.915 ± 0.30 mM in the first 2 h (Table 1). The control strain did not produce a significant amount of VA, again supporting the hypothesis that the expression of native ATAs is not induced under the applied conditions.

After the initial production phase lasting for 2–7 h, a significant re‐assimilation of the product and a small amount of vanillyl alcohol and vanillic acid were observed in the broth during the bioconversion using a resting cell setup (Fig. S6). Vanillyl alcohol was the main by‐product for all strains excluding TMB‐JH004 (CV‐ATA), which did not produce significant amounts of by‐products (Fig. S6D). The lowest concentration was obtained by TMB‐NM0016 (0.280 ± 0.028 mM) and highest by the cells carrying the empty plasmid (0.490 ± 0.057 mM). In contrast, the bioconversion of VA using GN442ΔPP_2426 produced mainly vanillic acid as the main by‐product ant its production started between 6–10 h for all strains overexpressing ATA with activity against VA (Fig. S5). From this, it is clear that GN442 ΔPP_2426 still carries endogenous dehydrogenase activity towards vanillin. Apparently, the ATA‐based conversion of vanillin to VA competes with remaining vanillin dehydrogenase background activity converting it to by‐products and further metabolization. When the rate of VA formation is slowed down (after the initial 2‐7h production phase), the reaction equilibrium shifts and VA is re‐assimilated. It is probably connected to a very low amount of vanillin in the cell driving the equilibrium towards vanillin production from VA and further conversion to by‐products, especially when glucose is added to the broth (Fig. S5).

Worth noting, however, is that only a fraction of the original vanillin was converted, indicating that resting cells may be suitable to avoid assimilation via vanillic acid and vanillyl alcohol due to limited regeneration of NAD(P)^+^under the applied conditions. Supporting this claim is that the vanillin was fully converted when the strains were grown in M9 medium supplemented with glucose (Fig. S5). Another conclusion is that the transamination reaction equilibrium was dynamic under the applied batch process configuration. Further metabolic engineering and optimization of reaction conditions to reduce the intracellular concentration of endogenous amine acceptors (e.g. pyruvate) may prolong the production phase.

### Effect of l‐alanine regeneration by AlaDH on whole‐cell bioconversion of vanillin to VA

**Fig. 4:**
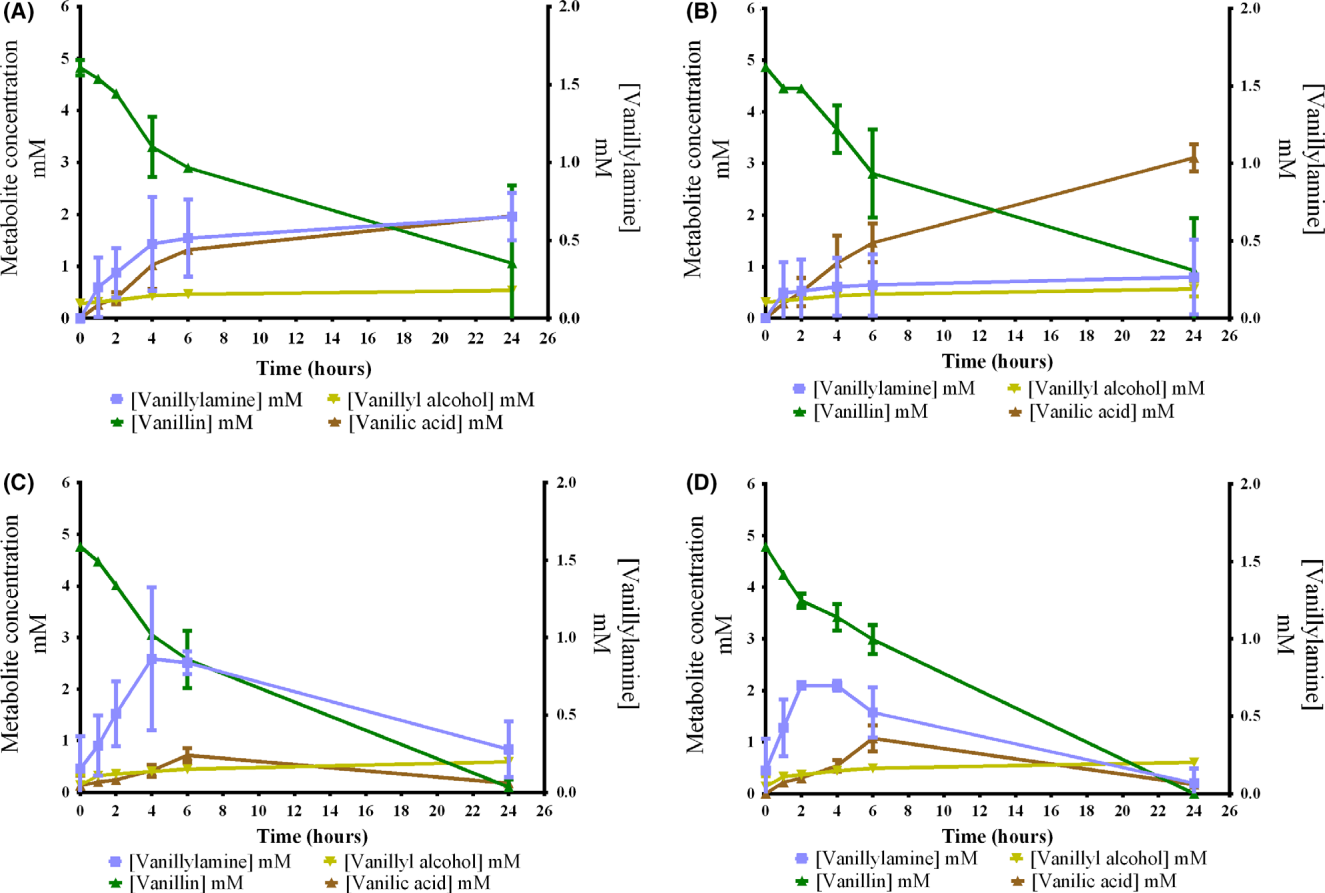
Effect of l‐alanine regeneration on whole‐cell bioconversion of vanillin to vanillylamine using metabolically engineered *P.putida* GN442ΔPP_2426 strains as biocatalysts. Bioconversions were performed with a high cell density (OD_620_ = 10) for 24 h at 30 °C and 180 rpm in M9 medium supplemented with 5 mM vanillin, 10 g/L of glucose for NADH regeneration and 200 mM of NH_4_Cl. l‐Alanine was omitted for A and B and 50 mM of l‐alanine was added to the medium for C and D. A and C: TMB‐JH006 (Cv‐ATA + AlaDH) and B and D: TMB‐JH004 (Cv‐ATA). Error bars indicate ± SD of two biological replicates.

The low titres obtained and re‐assimilation of the product could be a consequence of unfavourable reaction equilibrium resulting in vanillin oxidation to vanillic acid being the dominating activity. Supplementation of amine donor proved to be indispensable when the bioconversion was starting with low cell density (OD_620_ 0.3˜0.5) since, without supplementation, cells failed to produce VA (Fig. S7). However, even with supplementation, low intracellular availability of l‐alanine and inefficient pyruvate removal by endogenous enzymes may still disadvantage the desired reaction's direction. The introduction of AlaDH from *B*. *subtilis* for the regeneration of l‐alanine from pyruvate could assist in shifting the equilibrium, as previously shown for *E. coli* (Wu *et al*., 2016; Wu *et al*., 2017a; Patil *et al*., 2019). l‐alanine regeneration was investigated by cloning the *AlaDH* gene in the plasmid pJH004 and inserting it in GN442ΔPP_2426. The resulting strain was named TMB‐JH006 and overexpresses both *Cv‐ATA* and *Bs*‐*AlaDH,* under the same promoter control. Whole‐cell bioconversion of vanillin was performed in M9 medium supplemented with glucose since the AlaDH requires efficient NADH regeneration to convert pyruvate to l‐alanine. For this experiment, a high cell density (OD_620_ = 10) was used to enable higher volumetric productivity and minimize cell growth (Fig. 4). Also, the possibility to reach VA by supplementation of ammonium as the sole nitrogen source and to rely on AlaDH to produce l‐alanine intracellularly was investigated. Indeed, the amine donor omission did not abolish the transamination of vanillin to VA by TMB‐JH006 and TMB‐JH004 (Fig. 4A and B, respectively), and coexpression of AlaDH resulted in increased production regardless if l‐alanine was added or not.

In the absence of l‐alanine, the maximum titre of VA reached by TMB‐JH004 and TMB‐JH006 were 0.27 ± 0.24 mM and 0.65 ± 0.15 mM, respectively, while in the presence of the amine donor, TMB‐JH004 produced 0.70 ± 0.01 mM and TMB‐JH006 0.84 ± 0.07 mM of VA. In l‐alanine's presence, these values were obtained in the first 6 h of bioconversion, and after this point, the re‐assimilation of VA occurred. In contrast, VA re‐assimilation did not occur in the absence of the amine donor, and more VA could be detected in the broth after 24 h, especially for the cell overexpressing both *Cv‐ATA* and *AlaDH* (0.65 ± 0.15 mM versus 0.28 ± 0.18 mM, respectively). Therefore, the utilization of ammonium as the sole source of amine donors is efficient to avoid re‐assimilation of VA, although a lower amount of product is formed in the initial phase of bioconversion (6 first hours). Furthermore, in the presence of l‐alanine, the optimum sampling point appears to occur in 4 h of bioconversion, which precedes the re‐assimilation of VA. While in the absence of l‐alanine, a longer bioconversion time is advantageous for the transamination reaction. A more detailed profile regarding the *in vivo* transamination of vanillin could indicate the optimum harvest time, which may occur either between 6 and 24 h or after this. These results indicate that relying on endogenous amine donors, which can be supplied via the addition of ammonium, is an efficient strategy to prevent VA re‐assimilation.

The observed difference for experiments with and without external supplementation of l‐alanine may be due to endogenous transaminases background activity using other amine acceptors than vanillin as substrate and thereby increasing intracellular pyruvate levels from externally supplemented l‐alanine. Pyruvate formed by this route could then be available as an amine acceptor for conversion of VA to vanillin by Cv‐ATA or by additional native ATAs described in this study (Table 1). Although the pyruvate was not measured, it can be speculated that its intracellular levels are higher in the presence of l‐alanine, and this impacted VA production. Nevertheless, the results indicate that it is advantageous to use ammonium for two reasons, (i) since it could make the addition of expensive amine donors redundant, although its utilization increases considerably the efficiency of the bioconversion in the initial phase, and (ii) since it avoids product re‐assimilation.

Formation of the by‐products vanillyl alcohol and vanillic acid was higher in a growing‐cell setup compared with using resting cells without glucose in the medium (Fig. S6), explaining why all vanillin was converted in the first 24 h when metabolically active cells were utilized (Fig. 4). The presence of glucose is likely to have maintained redox homeostasis, that is the NAD+/NADH ratio, which probably is the explanation for the higher by‐product formation compared to the resting cell setup. Furthermore, a higher amount of cells was utilized in the bioconversion of vanillin by growing cells of TMB‐JH004 and TMB‐JH006 (OD_620_ = 10; Fig. 4) than the resting cells (OD_620_ = 3; Fig. S6), which may also have contributed to the higher amount of vanillic acid and vanillyl alcohol observed (Fig. 4B and D; and Fig. S6D).

Although the strain GN442ΔPP_2426 has significantly lower oxidoreductase activity towards vanillin compared to KT2440, other aldehyde dehydrogenases need to be deleted to increase the efficiency of vanillin biotransformation. In a previous proteomics study, the aldehyde dehydrogenases PP_3151, PP_5120 and PP_5258 showed an increased abundance in response to vanillin, representing possible targets to abolish the oxidation of vanillin into vanillic acid (Simon *et al*., 2014).

### Whole‐cell bioconversion of ferulic acid to VA

**Fig. 5:**
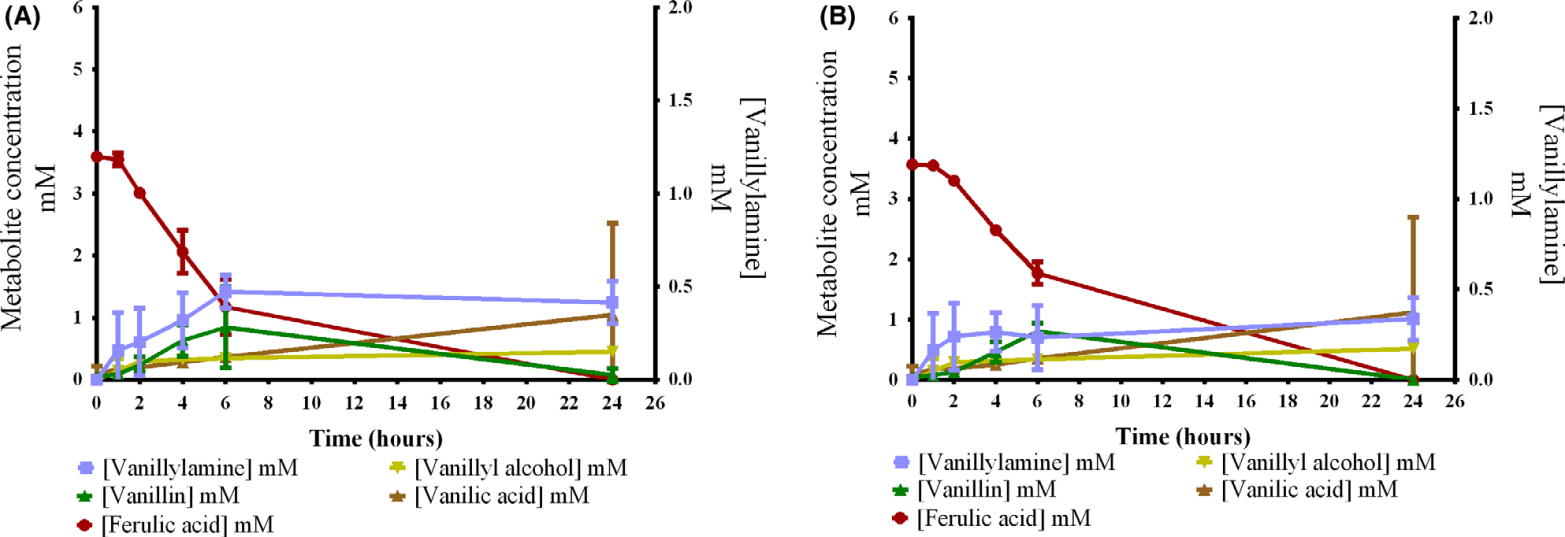
Whole‐cell bioconversion of ferulic acid to vanillylamine using metabolically engineered *P.putida* GN442ΔPP_2426 strains as biocatalysts. Bioconversions were performed with high cell density (OD_620_ = 10) for 24 h at 30 °C and 180 rpm in M9 medium supplemented with 3.5 mM ferulic acid, 10 g l^−1^ of glucose for NADH regeneration, 50 mM of l‐Alanine and 200 mM of NH_4_Cl. A: TMB‐JH006 strain (Cv‐ATA + AlaDH) and B: TMB‐JH004 strain (Cv‐ATA). Error bars indicate ± SD of two biological replicates.

An additional series of experiments to investigate whether it was possible to convert ferulic acid to vanillylamine were performed. Similar to vanillin bioconversion, a significant amount of ferulic acid was assimilated, and the by‐products vanillyl alcohol and vanillic acid were formed, resulting in a limited yield of VA (Fig. 5).

An increase of the VA production was observed in the strain coexpression Cv‐ATA and AlaDH (0.473 ± 0.09 mM compared to 0.235 ± 0.18 mM, p = 0.2738) in the first 6 h of bioconversion. In contrast to vanillin bioconversions with l‐alanine as amine donor, VA re‐assimilation was remarkably not observed for the ferulic acid bioconversions (Fig. 4C and D). This indicates that a reduced intracellular level of pyruvate in combination with higher levels of l‐alanine and/or additional endogenous metabolites acting as amine donors achieved the desired reaction equilibrium. Further investigation of intracellular metabolites under the reaction's progress is required to shed light on the matter. However, the possibility to use *P. putida* to reach VA from ferulic acid through whole‐cell bioconversion is clear.

## Conclusions

In this study, a novel method to select for recombinant vanillin transaminases was developed. The selection principle is based on the ability of *P. putida* KT2440 to utilize VA as the sole carbon source to sustain growth only if a suitable ATA is overexpressed. The method is easy‐to‐use and can be integrated into the transformation pipeline, thereby potentially being used for screening campaigns covering large sequence spaces. Here, the method was used to identify Pp‐SpuC‐II and Cv‐ATA to possess high *in vivo* activity when overexpressed in *P. putida*. Whole‐cell bioconversion of vanillin and ferulic acid to VA in batch mode was restricted by competing activity of endogenous enzymes producing vanillic acid, resulting in reduced yield and loss of product by re‐assimilation. This points towards the need to knock out additional genes encoding oxidoreductases to make ω‐transamination the dominating activity. When resting cells were used, the formation of by‐products was significantly reduced; however, no increase in VA titres was observed. Also, using ammonium as the sole nitrogen source, instead of external supplementation of l‐alanine, minimized the re‐assimilation after the initial amine production phase. Yet, further investigation of intracellular reactants accepted by the recombinant transaminases under the different reaction phases and any potential influences of endogenous metabolites is needed to shed additional light into possible ways to shift the adverse reaction equilibrium. Also, optimization of other influencing parameters, such as PLP concentration, enzyme levels, ammonium concentration and external supply of co‐substrates for NADH regeneration, are additional potential strategies for improving VA production.

## Experimental procedures

### Chemicals and enzymes

All reagents (vanillin, VA, vanillyl alcohol, vanillic acid, ferulic acid and l‐alanine) were of analytical grade and were purchased from Merck KGaA (Darmstadt, Germany). The antibiotics ampicillin and streptomycin and isopropyl‐β‐D‐thiogalactoside (IPTG) were purchased from Sigma‐Aldrich (St. Louis, MO, USA). Restriction enzymes, T4 DNA ligase and Phusion High‐Fidelity DNA Polymerase utilized for gene cloning were ordered from Thermo Fisher Scientific (Vilnius, Lithuania). All DNA oligonucleotides (primers) utilized for cloning and sequencing were synthesized by Eurofins Genomics (Ebersberg, Germany).

### Molecular biology procedures

All procedures in the cloning steps were performed according to the manufacturer's recommendations (Thermo Fisher Scientific). Plasmid DNA was isolated using GeneJET Plasmid Miniprep Kit from Thermofisher (Vilnius, Lithuania). Agarose gel extractions were performed using GeneJET Gel Extraction Kit from the same manufacturer. Genomic DNA extraction from *P. putida* KT2440 was performed using the GeneJET Genomic DNA Purification Kit from Thermo Fisher Scientific Baltics (Vilnius, Lithuania). Primers utilized for polymerase chain reactions (PCRs) are described in the Table S1. Phusion High‐Fidelity DNA Polymerase was utilized for cloning and sequencing while DreamTaq Polymerase (Thermo Scientific, Germany) was utilized for colony PCR. GeneAmp PCR System 9700 (Applied Biosystems, Foster City, CA, USA) was used for the PCRs amplifications and the products were purified using GeneJET PCR Purification Kit (Thermofisher). Sequencing was performed with the amplified DNA fragments to verify the absence of mutations in the RBS sequence or coding sequence (Eurofins Genomics, Ebersberg, Germany).

### Plasmids and bacterial strains

All plasmids and strains utilized in this work are described in Tables S2 and S3, respectively. The pSEVA424 plasmid was obtained from the Standard European Vector Architecture (SEVA) repository (Silva‐Rocha *et al*., 2013) and it was utilized as the backbone for the construction of the plasmids pJH001 (*Cc‐ATA*), pJH002 (*Cv‐ATA*), pJH004 (*Cv‐ATA + AlaDH*), pNM011 (*Pp‐SpuC‐II*), pNM012 (*Pp‐SpuC‐I*) and pNM013 (*Pp‐ATA*). The *AlaDH* gene (Sequence S1) from *B. subtilis* 168 (Genbank ID: 936557, Swiss‐Prot: Q08352) was synthesized by GenScript (New Jersey, USA) and delivered in plasmid pUC57‐AlaDH. *Cc‐ATA* gene encoding vanillin transaminase from *C. chinense* (GenBank: AAC78480.1, Swiss‐Prot: O82521) and *Cv‐ATA* from *C. violaceum* (GenBank: WP011135573.1, Swiss‐Prot: Q7NWG4) were amplified from pNW10 and pNW12 (Weber *et al*., 2017), respectively (primers described in Table S4). Genes encoding the native ATAs Pp‐SpuC‐II (GenBank: AAN70747.1), Pp‐SpuC‐I (GenBank: AAN67793.1) and Pp‐ATA (GenBank AAN68196.1) were amplified from *P. putida* KT2440 genome. PCR products were digested and ligated into backbone plasmid pSEVA424 digested with compatible restriction enzymes (Table S4). To construct the plasmid containing the *AlaDH* gene, the synthetic pUC57‐AlaDH was digested with the restriction enzymes *PsTI* and *SpeI*. The resulting fragment was inserted into pJH002 digested with the same restriction enzymes, creating pJH004. The consensus Ribosomal Binding Site (RBS) sequence and the eight conserved nucleotides downstream to this sequence were added immediately upstream to the start codon of the *AlaDH* coding sequence (Silva‐Rocha *et al*., 2013). *Escherichia coli* DH5α strain was utilized for subcloning and plasmid maintenance. *P. putida* KT2440 (DSM 6125) was obtained from German Collection of Microorganisms and Cell Cultures (D.S.M.Z.; Braunschweig, Germany). The engineered strain GN442ΔPP_2426 was constructed previously (García‐Hidalgo *et al*., 2020) utilizing GN442 strain (Graf and Altenbuchner, 2014) as the scaffold. KT2440 was utilized as background for the construction of TMB‐JH001, TMB‐JH002, TMB‐NM011, TMB‐NM012 and TMB‐NM013 strains, while GN442ΔPP_2426 strain was used to construct the other strains (Table 1). All bacterial strains were prepared and kept in glycerol stocks 20% at −80 °C until the experiments.

### Electrocompetent cells preparation and transformation conditions

Heat‐shock transformation of *E. coli* was performed as described previously (Green and Sambrook, 2018). Transformed cells were selected in LB plates supplemented with ampicillin (100 µg ml^−1^) or streptomycin (60 µg ml^−1^). *P. putida* KT2440 and GN442ΔPP_2426 electrocompetent cells were prepared as described previously (Martínez‐García and de Lorenzo, 2012) with slight modifications. Overnight cultures were transferred to a fresh LB medium with the OD_620_ adjusted to 0.1 and then cultivated in shake flasks until OD_620_ reached 0.8. Cells in mid‐exponential‐phase were washed two times with sucrose (300 mM) and re‐suspended in the same solution. Freshly prepared electrocompetent cells and plasmids (100 ng) were mixed in an electroporation cuvette with 2 mm gap width. The electric pulse was applied using a Gene Pulser apparatus equipped with a Pulse Controller (Bio‐Rad, Hercules, CA, USA). Subsequently, cells were plated on M9 plates with citrate (10 mM) or vanillylamine (5 mM) as sole carbon source and streptomycin (100 μg/ml) for the selection. For the growth‐based ATA selection method, 1 mM IPTG was added to induce gene expression. Cells were diluted 1000x and 10 000x using a sodium chloride solution (0.9% NaCl) prior to plating. Plates were incubated at 30 °C for 3 days.

### Media and cultivation conditions

Lysogeny broth (LB) medium (tryptone 10 g l^−1^, yeast extract 5 g l^−1^, NaCl 10 g l^−1^) was used for routine cultivation of *E. coli* strain DH5α at 37 °C and *P. putida* strains at 30 °C. M9 medium (Sambrook and Russell, 2001; 6 g l^−1^ disodium phosphate, 3 g l^−1^ monopotassium phosphate, 0.5 g l^−1^ sodium chloride, 1 g l^−1^ ammonium chloride, 2 mM magnesium sulphate, 100 μM calcium chloride, pH 7) with 1% (v/v) trace elements solution 100× (0.5 g l^−1^ EDTA disodium dihydrate, 0.2 g l^−1^ FeSO_4_ ∙ 7 H_2_O, 0.01 g l^−1^ ZnSO_4_ ∙ 7 H_2_O, 0.003 g l^−1^ MnCl_2_ ∙ 4 H_2_O, 0.03 g l^−1^ H_3_BO_3_, 0.02 g l^−1^ CoCl_2_ ∙ 6 H_2_O, 0.001 g l^−1^ CuCl_2_ ∙ 2 H_2_O, 0.002 g l^−1^ NiCl_2_ ∙ 6 H_2_O, 0.003 g l^−1^ Na_2_MoO_4_ ∙ 2 H_2_O; Pfennig and Lippert, 1966) and appropriate carbon source was used for cultivation of *P*. *putida*. Stock solutions of VA 10× (50 mM), vanillin 10× (50 mM), d‐glucose 10× (100 g l^−1^) and citrate 50× (0.5 M) were sterilized by filtration using 0.2 μm filter and added to the M9 media immediately before use. Ampicillin was utilized for *E*. *coli* selection (100 µg ml^−1^) and streptomycin in the selection of *E*. *coli* (60 µg ml^−1^) as well for *P. putida* strains (100 µg ml^−1^). IPTG was added to induce the expression of ATA genes regulated by promoter system *lacIq*‐P*trc*. To evaluate the capacity of *P. putida* KT2440 to metabolize VA, one single colony was picked in a LB plate and grown overnight on M9 medium with glucose (10 g l^−1^). On the following day, the pre‐inoculum was washed with a sodium chloride solution (0.9% NaCl) to remove residual glucose. Optical density at 620 nm (OD_620_) was measured using a Ultrospec 2100pro spectrophotometer (Amersham Bioscences, Sweden) to prepare the inoculum (initial OD_620_ = 0.3) at 250 ml baffled shake flask containing 25 ml of M9 medium supplemented with 5 mM vanillylamine, 5 mM vanillin or 10 g l^−1^ d‐glucose (55 mM) as carbon source. Cells were cultivated for 48 h at 30 °C and under shaking (180 rpm) using a Innova 43 Shaking Incubator (New Brunswick Scientific, Edison, NJ, USA) and the OD_620_ was monitored periodically. All shake flask cultivations were carried out in three biological replicates. For the plate reader growth experiments, an individual colony was used to inoculate 5 ml of LB containing 10 μg ml^−1^ of streptomycin. Overnight culture was then washed twice in M9 medium and diluted to a final OD of ˜0.25–0.4 into a 96‐well microtitre plate (Sarstedt, Germany) supplemented with vanillylamine ranging from 0 to 25 mM. Plates were incubated in a Multiskan™ FC Microplate Photometer (Thermo Fisher Scientific, USA) plate reader at 30 °C with shaking before each measurement of OD_620_ for a time period of 24 h. Growth curves were fitted to a logistic curve (Equation 1) using Python SciPy Optimize (Oliphant, 2007).

(1) $$f\left(t\right)=\frac{K}{1+\left(\;\frac{K‐x_{0}}{x_{0}}\right)\cdot e^{‐rt}}+b,$$

where *K* is the carrying capacity, *r* is the growth rate, *x*_0_ is the initial cell concentration, *t* is the time and *b* is the floor term (accounting for measurement background).

### Whole‐cell bioconversions

*Pseudomonas putida* strains were pre‐grown overnight at 30 °C with shaking in 10 ml LB supplemented with streptomycin to avoid plasmid loss. Subsequently, cells were harvested by centrifugation (4000 rpm, 5 min), washed and transferred to M9 media supplemented with 1 mM IPTG, 100 µg ml^−1^ streptomycin and either 10 g l^−1^ glucose, 5 mM vanillylamine, 5 mM vanillin, in a 250 ml baffled shake flask to an OD of ˜0.3–0.5. For preparation of cells for bioconversions using high cell density (OD_620_ = 3–10 as indicated in the results and discussion), the pre‐cultures were instead grown in 50–100 ml LB supplemented with streptomycin in a 500 ml baffled shake flask and grown until OD ˜ 1. At this point, 5 mM IPTG was added to induce gene expression, and the culture was grown at 23 °C overnight. Subsequently, cells were harvested and washed, and re‐suspended in 5 ml sodium phosphate buffer (50 mM, pH 7.2) or 5 ml M9 medium supplemented with 5–10 mM vanillin or 3–10 mM ferulic acid, 0–10 g l^−1^ glucose, 0–100 mM l‐alanine and/or 0‐200 mM of ammonium chloride. Whole‐cell bioconversions were performed at 30 °C under aerobic conditions in shake flasks or glass vials with stirring for 24 h, and 0.5 ml samples were frequently withdrawn for OD620 measurements, centrifuged and frozen at −20 °C prior to HPLC analysis.

### HPLC analysis

For HPLC metabolite analysis, a Select C18 column (4.6 × 150 mm) was used with a Waters HPLC system (Waters Binary HPLC pump 1525, UV/Vis detector 2489, Auto sampler 2707, All Waters Corporation, Milford, USA). Reverse‐phase chromatography was performed using two mobile phases: millipore water with 0.1% trifluoroacetic acid (TFA; A) and acetonitrile (B). An isocratic method with 65% A and 35% B for 10 min with a flow of 1 ml min^−1^, at a monitored wavelength at 281 nm was used. The analysis was performed at room temperature. A standard curve was made to calculate the concentration of the product in the sample.

### Data analysis

All bioconversions curves graphs were prepared using Graphpad Prism software version 6.0 (GraphPad Software, San Diego, CA, USA) and display the concentration of metabolites obtained from HPLC analysis and/or the OD620 measured by spectrophotometer. The error bars represent the standard deviation from at least two biological replicates. The statistical analyses were performed using Graphpad Prism software (version 6.0). The one‐way ANOVA test followed by Tukey's post‐test (*P* < 0.05) or the unpaired *t*‐test with Welch's correction (*P* < 0.05) were performed when three and two samples were compared, respectively.

## Conflict of interest

The authors declare that they have no conflict of interest.

## Author contributions

J.H.C.M.N. contributed to the design of the study, performed the bioinformatics analysis, design and performed the experiments, analysed the data and drafted the manuscript. F.L. and N.M. helped to perform the bioconversions experiments, the growth curves and helped with the data interpretation and write the paper. N.M. developed the HPLC setup used and helped with the analytical analyses, the data interpretation and to write the Experimental Procedures section. E.M.L. performed the growth curves in microplates and prepared the parameter fitting for the growth curves, helped to perform the bioconversions experiments and write the paper. N.S.P. and MC revised and helped to write the manuscript. MC conceived and designed the study and participated in the experimental design and data interpretation. All authors read and approved the final manuscript.

## Supporting information


**Fig. S1.** Growth profiles of *P. putida* KT2440 cultivated for 48 hours at 30°C and 180 rpm in shake flasks with 50 ml M9 medium. Vanillin, vanillylamine and glucose were utilized as carbon sources. Error bars indicate ± SD of three biological replicates.
**Fig. S2.** Growth profiles of *P. putida* KT2440 overexpressing ATAs encoding genes on M9 medium supplemented with a range of vanillyalmine concentrations (0‐25 mM).
**Fig. S3.** Whole‐cell bioconversion of vanillylamine using growing‐cells of *P. putida* KT2440 strains over‐expressing different ATAs encoding genes.
**Fig. S4.** Effect of the antibiotic in the selection of positive clones containing *in vivo* transaminase activity against vanillylamine.
**Fig. S5.** Whole‐cell bioconversion of vanillylamine using growing‐cells of metabolically engineered *P. putida* GN442ΔPP_2426 strains over‐expressing different ATAs encoding genes.
**Fig. S6.** Whole‐cell bioconversion of vanillin to vanillylamine using resting‐cells of metabolically engineered *P.putida* GN442ΔPP_2426 strains over‐expressing different ATAs encoding genes.
**Fig. S7.** Whole‐cell bioconversion of vanillin to vanillylamine without amine donor using growing‐cells of metabolically engineered *P. putida* strains as *P.putida* GN442ΔPP_2426 strains over‐expressing different ATAs encoding genes.
**Fig. S8.** SDS‐PAGE gel from cell crude extract of GN442ΔPP_2426 strains overexpressing different ATA encoding genes.

**Table S1.** BLASTp results for each ATA utilized as query sequences against *P. putida* KT2440 database.
**Table S2.** List of plasmids used in this work.
**Table S3.** List of strains used in this work.
**Table S4.** List of PCR primers utilized in this work.
**Sequence S1.** Nucleotide sequence of the *AlaDH* gene from *B. subtilis* 168.
