# Supplementary material for: Metabolic engineering of Pseudomonas putida for production of vanillylamine from lignin‐derived substrates
Source: Microb Biotechnol. 2021 Feb 3;14(6):2448–62. doi: 10.1111/1751-7915.13764 (PMC8601178; doi:10.1111/1751-7915.13764)

Metabolic engineering of *Pseudomonas putida* for production of vanillylamine from lignin-derived substrates

João Heitor Colombelli Manfrão-Netto^1^, Fredrik Lund^2^, Nina Muratovska^2^, Elin M. Larsson^2,3^, Nádia Skorupa Parachin^1^, Magnus Carlquist^2,*^

^1^Grupo Engenharia de Biocatalisadores, Instituto de Ciências Biológicas, Universidade de Brasília, Brasília-DF, Brazil

^2^Division of Applied Microbiology, Department of Chemistry, Faculty of Engineering, Lund University, PO Box 124, 221 00 Lund, Sweden

^3^Department of Bioengineering, California Institute of Technology, 1200 East California Blvd, Pasadena, CA, 91125, USA

*Correspondence: [magnus.carlquist@tmb.lth.se](mailto:magnus.carlquist@tmb.lth.se)

**Figure S1.** Growth profiles of *P. putida* KT2440 cultivated for 48 hours at 30 °C and 180 rpm in shake flasks with 50 ml M9 medium. Vanillin, vanillylamine and glucose were utilized as carbon sources. Error bars indicate ± SD of three biological replicates.

**Figure S2.** Growth profiles of *P. putida* KT2440 overexpressing ATAs encoding genes on M9 medium supplemented with a range of vanillyalmine concentrations (0-25 mM).

**Figure S3.** Whole-cell bioconversion of vanillylamine using growing-cells of *P. putida* KT2440 strains over-expressing different ATAs encoding genes.

**Figure S4.** Effect of the antibiotic in the selection of positive clones containing *in vivo* transaminase activity against vanillylamine.

**Figure S5.** Whole-cell bioconversion of vanillylamine using growing-cells of metabolically engineered *P. putida* GN442ΔPP_2426 strains over-expressing different ATAs encoding genes.

**Figure S6.** Whole-cell bioconversion of vanillin to vanillylamine using resting-cells of metabolically engineered *P.putida* GN442ΔPP_2426 strains over-expressing different ATAs encoding genes.

**Figure S7.** Whole-cell bioconversion of vanillin to vanillylamine without amine donor using growing-cells of metabolically engineered P. putida strains as *P.putida* GN442ΔPP_2426 strains over-expressing different ATAs encoding genes.

**Figure S8**.SDS-PAGE gel from cell crude extract of GN442ΔPP_2426 strains overexpressing different ATA encoding genes.

**Figure S1**. Growth profiles of *P. putida* KT2440 cultivated for 48 hours at 30 °C and 180 rpm in shake flasks with 50 ml M9 medium. Vanillin, vanillylamine and glucose were utilized as carbon sources. Error bars indicate ± SD of three biological replicates.


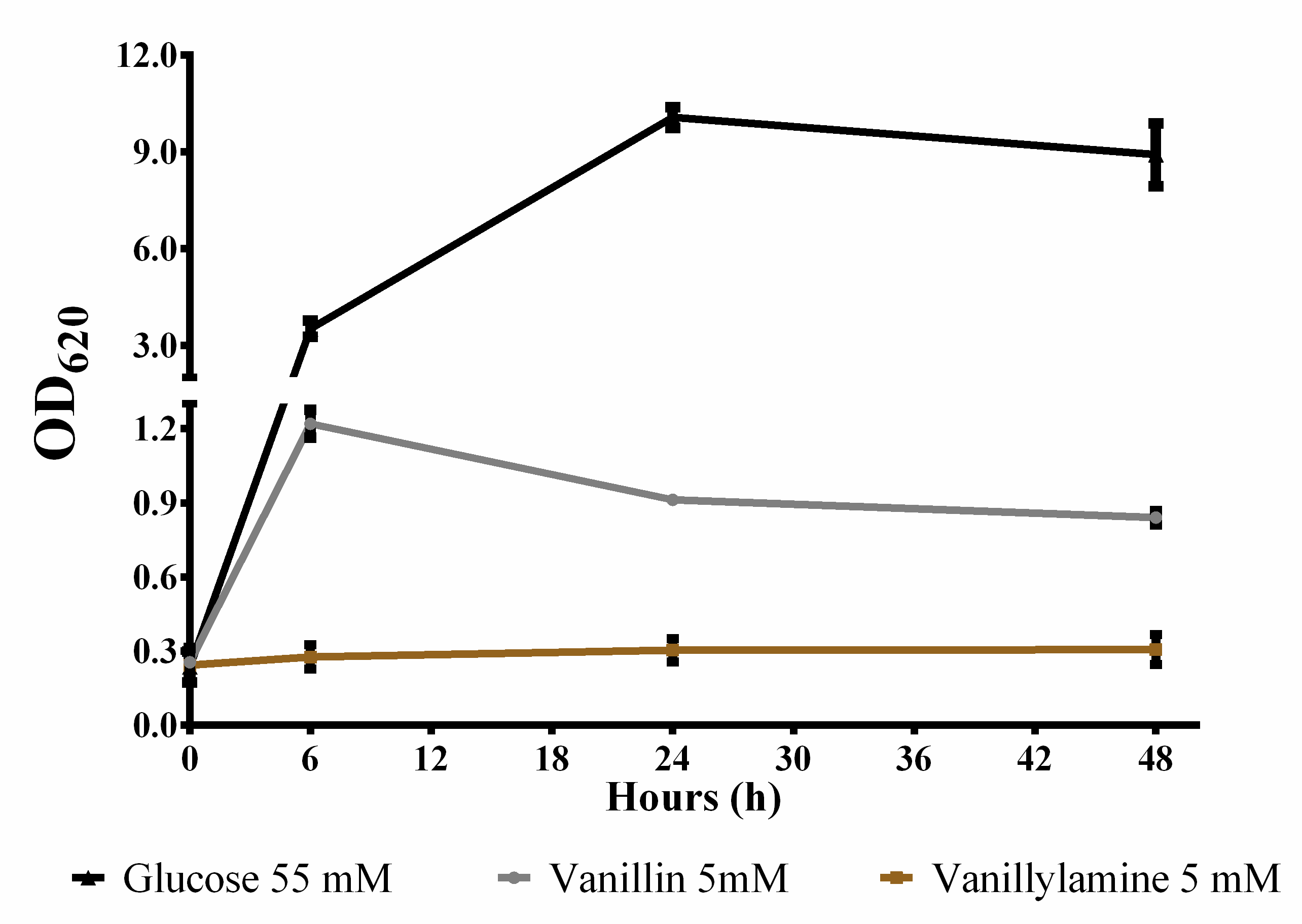


**Figure S2**. Growth profiles of *P. putida* KT2440 overexpressing ATAs encoding genes *P. putida* KT2440 wild-type (A), TMB-NM012 (*Pp-SpuC*-I) (B), and TMB-NM013 (*Pp-ATA*) (C), displayed no significant growth in M9 medium supplemented with a range of vanillylamine concentrations (0-25 mM) as the sole carbon source for 24 hours at 30C. In a pilot experiment performed in shake flasks, the strain overexpressing *Cc-ATA* showed no significant growth on 5 mM of VA as the sole carbon source and it was not used for microplate assays.


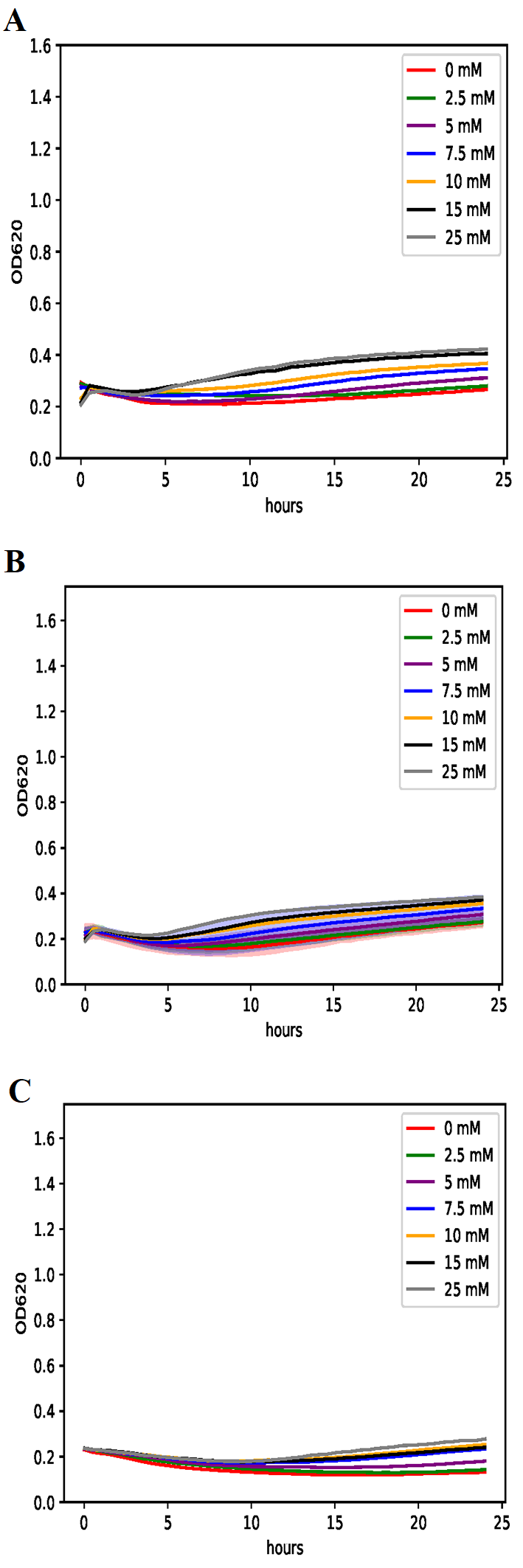


**Figure S3.** Whole-cell bioconversion of vanillylamine using growing-cells of *P. putida* KT2440 strains over-expressing (A) TMB-NM011 (Pp-SpuC-II), (B) TMB-NM012 (Pp-SpuC-I), (C) TMB-NM013 (Pp-ATA), (D) TMB-JH001 (Cc-ATA), (E) TMB-JH002 (Cv-ATA) or (F) Wild-type KT2440 (negative control). The bioconversions were performed for 24 hours at 30 °C and 180 rpm in M9 medium supplemented with 5 mM vanillylamine, 10 g/L glucose and 1 mM IPTG. For the Wild-type strain streptomycin was omitted. Error bars indicate ± SD of two biological replicates.

**
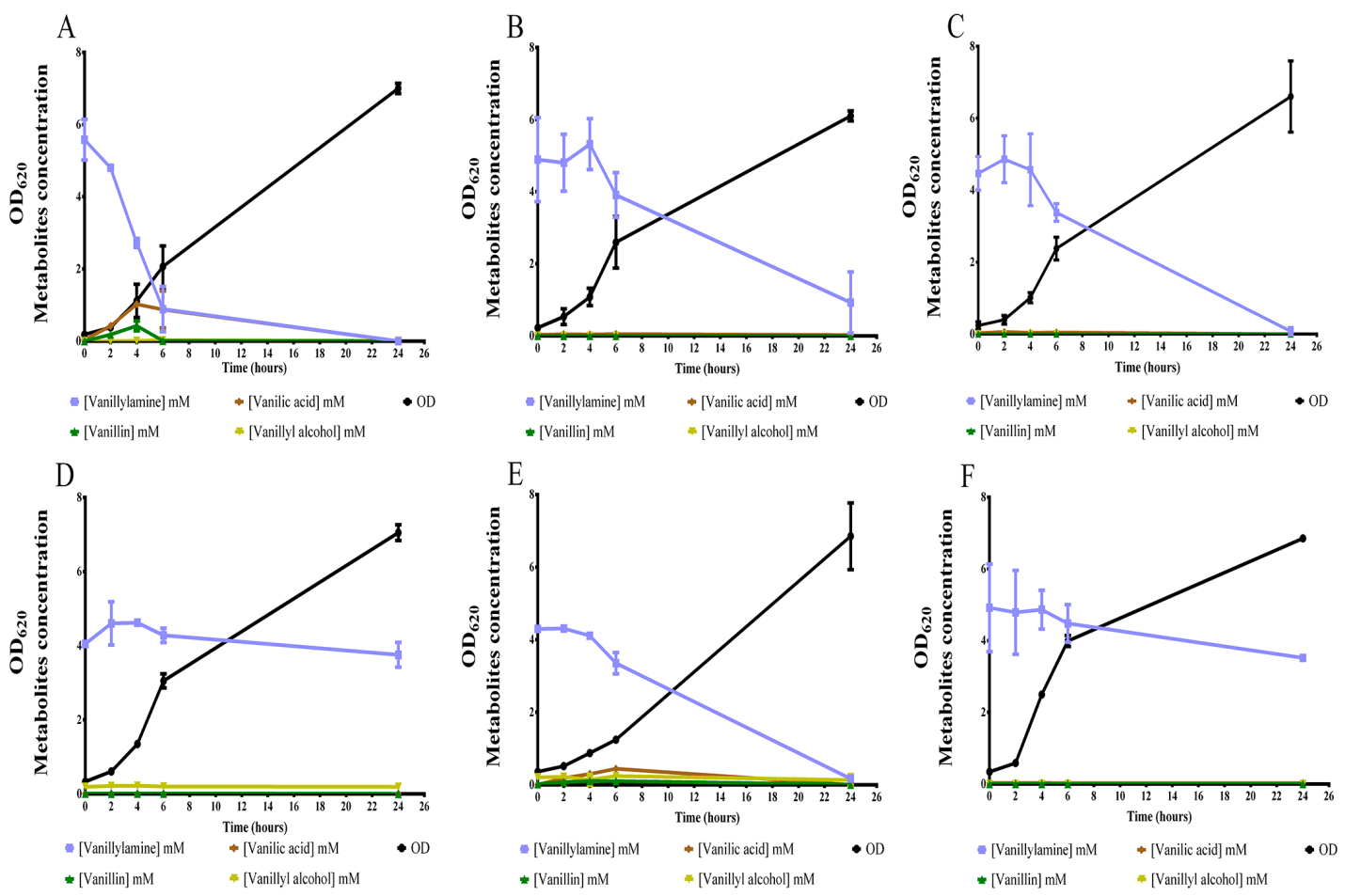
**

**Figure S4**. M9 plates after 3 days incubated at 30° C with 5 mM of VA as the sole carbon source and IPTG (1 mM) to induce the *Cv-ATA* gene expression. Streptomycin (100 µg/ml) was added to the plates in A and B while C and D only VA was utilized for the selection. A and C: *P. putida* KT2440 WT without pSEVA424 plasmid. B and D cells, TMB-JH002 cells expressing *Cv-ATA* gene.


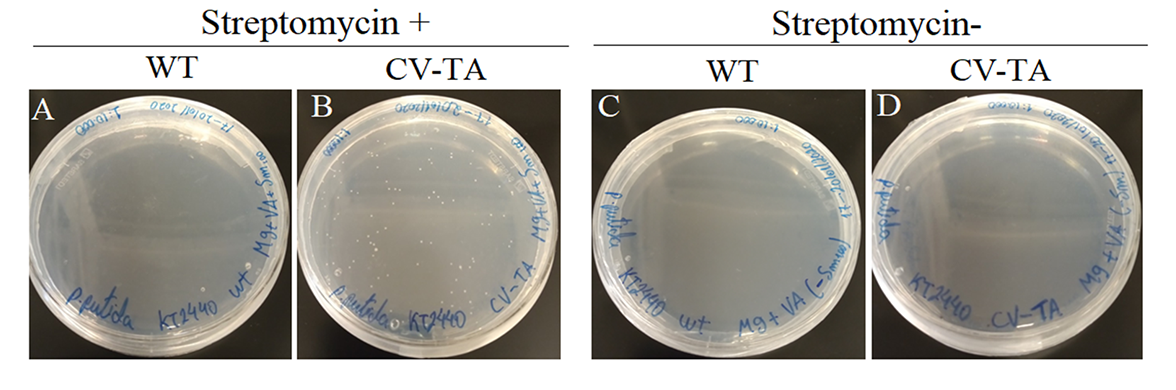


**Figure S5.** Whole-cell bioconversion of VA to vanillin using growing-cells of metabolically engineered *P.putida* GN442ΔPP_2426 strains as biocatalysts. The cells were cultured for 24 hours at 30 °C and 180 rpm in M9 medium with 5 mM of vanillylamine as the substrate for the bioconversion and 10 g/L of glucose as the carbon source. IPTG (1 mM) was added to induce the genes expression and 100 µg/ml streptomycin was added for plasmid maintenance. (A) TMB-NM014 (Pp-SpuC-II), (B) TMB-NM015 (Pp-SpuC-I), (C) TMB-NM016 (Pp-ATA),(D) TMB-JH003 (Cc-ATA), (E) TMB-JH004 (Cv-ATA) and (F) *P. putida* GN442ΔPP_2426 harboring the empty plasmid. This experiment was performed in two biological replicates, excluding TMB-JH003 (Cc-ATA).


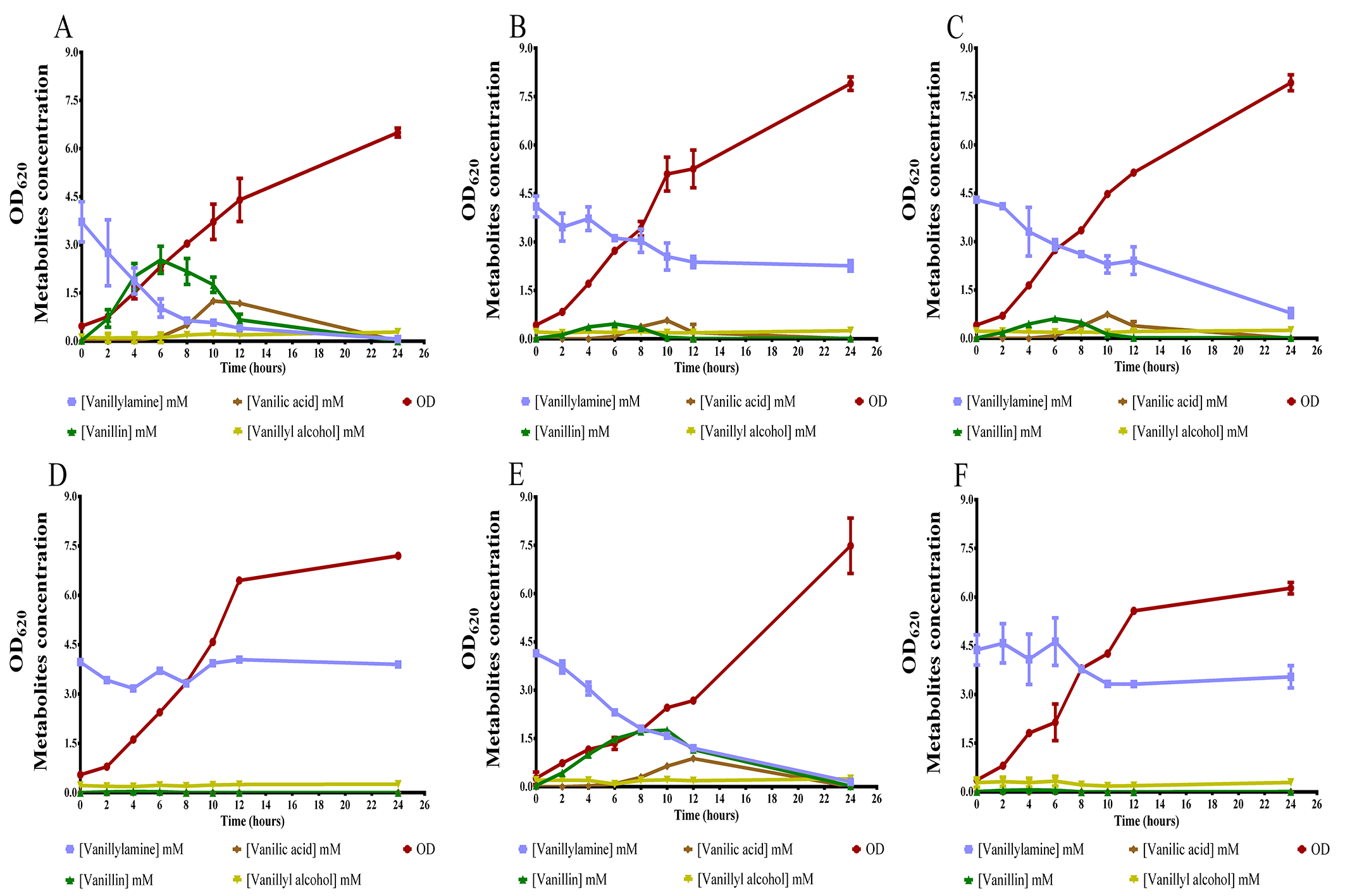


**Figure S6.**Whole-cell bioconversion of vanillin to vanillylamine using resting-cells of metabolically engineered *P.putida* GN442ΔPP_2426 strains as biocatalysts (OD_620_ =3). The assays were performed for 24 hours at 30 °C and 180 rpm using sodium phosphate buffer without the addition of glucose. Vanillin (10 mM) was utilized as the substrate and 100 mM of alanine as the amine donor. (A) TMB-NM014 (Pp-SpuC-II), (B) TMB-NM015 (Pp-SpuC-I), (C) TMB-NM016 (Pp-ATA),(D) TMB-JH004 (Cv-TA) and (E) *P. putida* GN442ΔPP_2426 harboring the empty plasmid. Error bars indicate ± SD of two biological replicates. **F.** Correlation between Specific transaminase activity* and maximum production of VA from vanillin.

*Specific activity was quantified by following the initial rate of whole-cell conversion of vanillylamine in M9 medium supplemented with glucose, and in turn is directly correlated with cell growth with VA as sole carbon source.


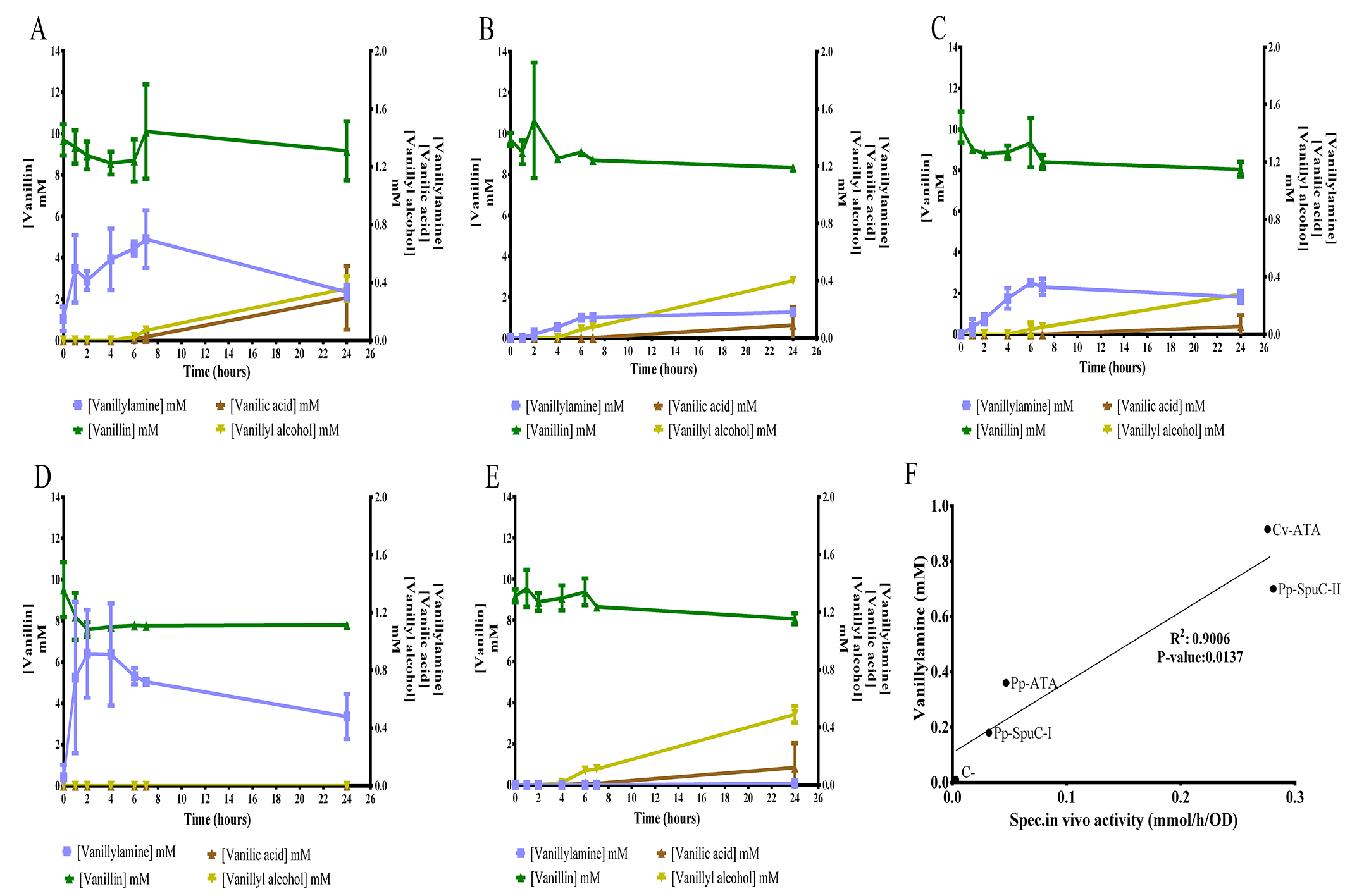


**Figure S7.** Whole-cell bioconversion of vanillin to VA without amine donor using growing-cells of metabolically engineered *P. putida* strains as biocatalysts. The cells were cultured for 24 hours at 30 °C and 180 rpm in M9 medium with 5 mM of vanillin as the substrate for the bioconversion and 10 g/L of glucose as the carbon source. IPTG (1 mM) was added to induce the genes expression, 100 µg/ml streptomycin was added for plasmid maintenance and the amine donor was omitted. (A) TMB-NM014 (Pp-SpuC-II), (B) TMB-NM015 (Pp-SpuC-I) or (C) TMB-NM016 (Pp-ATA), (D) TMB-JH004 (Cv-TA) and (E) *P. putida* GN442ΔPP_2426 harboring the empty plasmid. This experiment was performed in a single biological replicate.


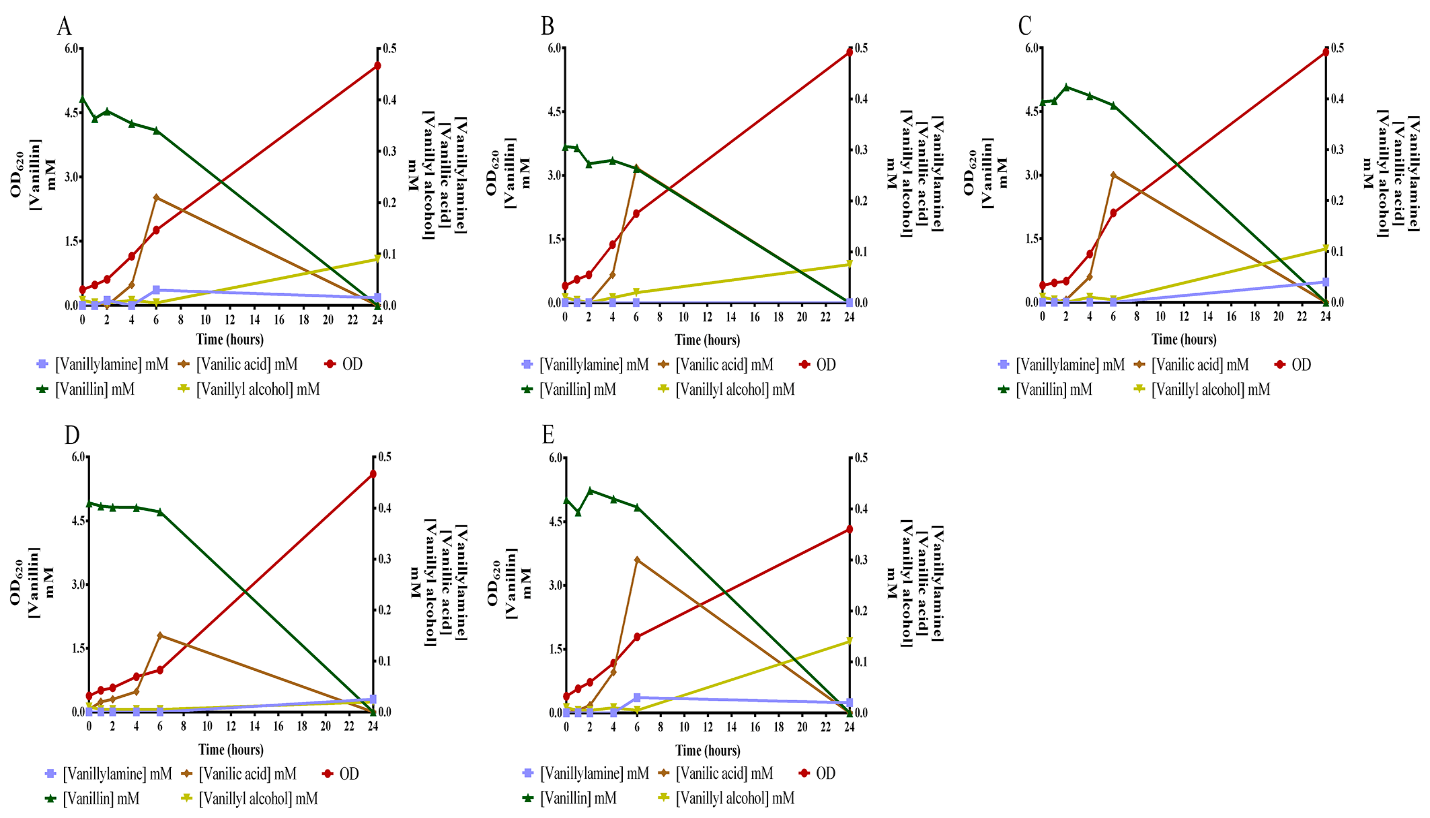


**Figure** S8. The pattern of proteins expressed by GN442ΔPP_2426 was assessed by SDS-PAGE analysis using cell crude extract. Pellets from bioconversion of VA to vanillin using growing cells of GN442ΔPP_2426 (experiment reported in Figure S5) after 24h were collected and frozen at -80 C. The proteins were extracted with YPER. Bradford assay was performed at 595 nm in a microplate and the concentration of total protein was estimated using a standard curve from BSA. Sample volume for SDS-PAGE was calculated so that 10µg of protein was loaded for each sample onto a 4-20% polyacrylamide Mini-PROTEAN TGX gel. (A) TMB-NM014 (Pp-SpuC-II; Mass: 49.88 kDA), (B) TMB-NM015 (Pp-SpuC-I; Mass: 49.64 kDA), (C) TMB-NM016 (Pp-ATA, Mass: 50.86 kDA),(D) TMB-JH003 (Cc-ATA, Mass: 50.74 kDA), (E) TMB-JH004 (Cv-ATA; Mass: 51.22 kDA) and (F) *P. putida* GN442ΔPP_2426 harboring the empty plasmid.


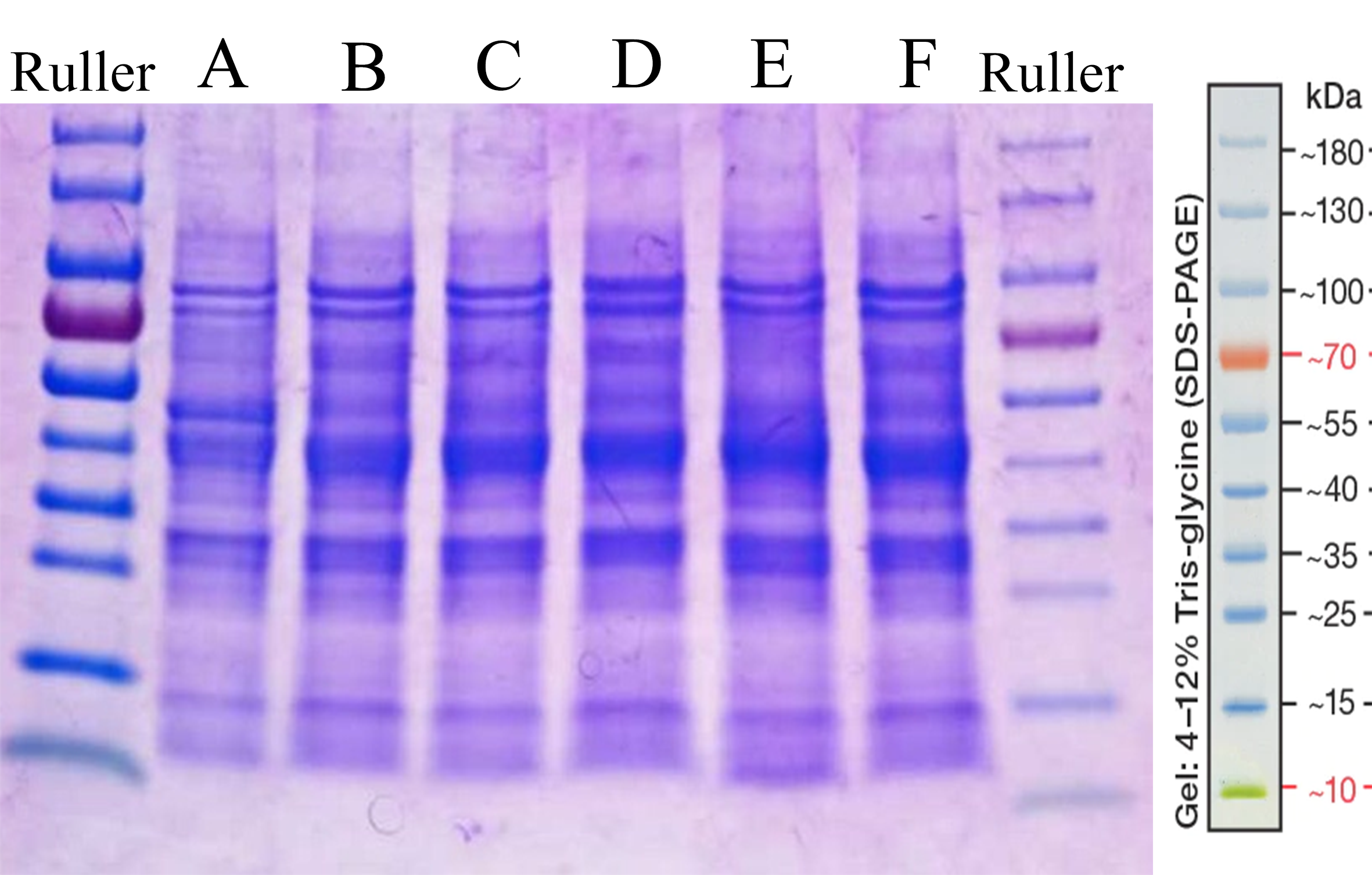

Supplement: Supplementary file 1 — Fig. S1. Growth profiles of P. putida KT2440 cultivated for 48 hours at 30°C and 180 rpm in shake flasks with 50 ml M9 medium. Vanillin, vanillylamine and glucose were utilized as carbon sources. Error bars indicate ± SD of three biological replicates. Fig. S2. Growth profiles of P. putida KT2440 overexpressing ATAs encoding genes on M9 medium supplemented with a range of vanillyalmine concentrations (0‐25 mM). Fig. S3. Whole‐cell bioconversion of vanillylamine using growing‐cells of P. putida KT2440 strains over‐expressing different ATAs encoding genes. Fig. S4. Effect of the antibiotic in the selection of positive clones containing in vivo transaminase activity against vanillylamine. Fig. S5. Whole‐cell bioconversion of vanillylamine using growing‐cells of metabolically engineered P. putida GN442ΔPP_2426 strains over‐expressing different ATAs encoding genes. Fig. S6. Whole‐cell bioconversion of vanillin to vanillylamine using resting‐cells of metabolically engineered P.putida GN442ΔPP_2426 strains over‐expressing different ATAs encoding genes. Fig. S7. Whole‐cell bioconversion of vanillin to vanillylamine without amine donor using growing‐cells of metabolically engineered P. putida strains as P.putida GN442ΔPP_2426 strains over‐expressing different ATAs encoding genes. Fig. S8. SDS‐PAGE gel from cell crude extract of GN442ΔPP_2426 strains overexpressing different ATA encoding genes. [file MBT2-14-2448-s002.docx]
