# Supplementary material for: Metabolic engineering of Pseudomonas putida for production of vanillylamine from lignin‐derived substrates
Source: Microb Biotechnol. 2021 Feb 3;14(6):2448–62. doi: 10.1111/1751-7915.13764 (PMC8601178; doi:10.1111/1751-7915.13764)
Supplement: Supplementary file 2 — Table S1. BLASTp results for each ATA utilized as query sequences against P. putida KT2440 database. Table S2. List of plasmids used in this work. Table S3. List of strains used in this work. Table S4. List of PCR primers utilized in this work. Sequence S1. Nucleotide sequence of the AlaDH gene from B. subtilis 168. [file MBT2-14-2448-s001.docx]

Metabolic engineering of *Pseudomonas putida* for production of vanillylamine from lignin-derived substrates

João Heitor Colombelli Manfrão-Netto^1^, Fredrik Lund^2^, Nina Muratovska^2^, Elin M. Larsson^2,3^, Nádia Skorupa Parachin^1^, Magnus Carlquist^2,*^

^1^Grupo Engenharia de Biocatalisadores, Instituto de Ciências Biológicas, Universidade de Brasília, Brasília-DF, Brazil

^2^Division of Applied Microbiology, Department of Chemistry, Faculty of Engineering, Lund University, PO Box 124, 221 00 Lund, Sweden

^3^Department of Bioengineering, California Institute of Technology, 1200 East California Blvd, Pasadena, CA, 91125, USA

*Correspondence: [magnus.carlquist@tmb.lth.se](mailto:magnus.carlquist@tmb.lth.se)

**Table S1.** BLASTp results for each ATA utilized as query sequences against *P. putida* KT2440 database.

**Table S2.** List of plasmids used in this work.

**Table S3.** List of strains used in this work.

**Table S4.** List of PCR primers utilized in this work.

**Sequence S1.** Nucleotide sequence of the *AlaDH* gene from *B. subtilis* 168

Table S1 . BLASTp results for each ATA utilized as query sequences against the protein database for *P. putida* KT2440.

| **Query ATA** | **GenBank Accession number** | **Locus tag** | **Pp-ATAs** | **Query cover (%)** | **Identity (%)** | **E-value** | **Description** |
| --- | --- | --- | --- | --- | --- | --- | --- |
| Pp-SpuC  (PDB id: 6HX9) | AAN70747.1 | PP_5182 (*spuC-II*) | Pp-SpuC-II | 99 | 98.01 | 0.0 | Polyamine:pyruvate transaminase |
|  | AAN67793.1 | PP_2180 (*spuC-I*) | Pp-SpuC-I | 99 | 84.99 | 0.0 | Polyamine:pyruvate transaminase |
|  | AAN68196.1 | PP_2588 | Pp-ATA | 98 | 51.89 | 2e-170 | Aminotransferase, class III |
| Cv-ATA  (PDB id: 4BA5 | AAN70747.1 | PP_5182 (*spuC-II*) | Pp-SpuC-II | 96 | 57.82 | 0.0 | Polyamine:pyruvate transaminase |
|  | AAN67793.1 | PP_2180 (*spuC-I*) | Pp-SpuC-I | 97 | 56.05 | 0.0 | Polyamine:pyruvate transaminase |
|  | AAN68196.1 | PP_2588 | Pp-ATA | 94 | 54.23 | 1e-169 | Aminotransferase, class III |

**Table S2**.List of plasmids used in this work

| **Plasmid** | **Description*** | **Reference** |
| --- | --- | --- |
| pSEVA424 | Broad host range plasmid regulated by expression promoter system *lacIq*-P*trc* under IPTG induction; Resistance marker Sm; Replication oriV(RK2) | Silva-Rocha et al. 2013 |
| pUC57-AlaDH | pUC57-derived plasmid bearing the synthetic *ald* gene encoding AlaDH from *Bacillus subtilis* 168 strain | This study |
| pNW10 | Yeast plasmid bearing the *Cc-ATA* gene from *Capsicum chinense* | Weber et al., 2017 |
| pNW12 | Yeast plasmid bearing the *Cv-ATA* gene from *Chromobacterium violaceum* | Weber et al., 2017 |
| pJH001 | Constructed plasmid derived from pSEVA424 backbone containing *Cc-ATA* gene under IPTG control | This study |
| pJH002 | Constructed plasmid derived from pSEVA424 backbone containing *Cv-ATA* gene under IPTG control | This study |
| pJH004 | Constructed plasmid derived from pSEVA424 backbone containing both *Cv-ATA* and *AlaDH* gene under IPTG regulation | This study |
| pNM011 | Constructed plasmid derived from pSEVA424 backbone containing *Pp-SpuC-II* gene under IPTG regulation | This study |
| pNM012 | Constructed plasmid derived from pSEVA424 backbone containing  *Pp-SpuC-I* gene under IPTG regulation | This study |
| pNM013 | Constructed plasmid derived from pSEVA424 backbone containing  *Pp-ATA* gene under IPTG regulation | This study |

*Antibiotic marker: Sm: Streptomycin.

**Table S3**. List of strains used in this work

| **Strain** | **Description*** | **Sourve or reference** |
| --- | --- | --- |
| *Escherichia coli* DH5α | Strain for plasmid maintenance and replication  F- ϕ*dlacZ* Δ*M*15 Δ*lacZYA*-*argF U169 deoR supE44 hsdR17 recA1 endA1 gyrA96 thi*-*1 relA1* | Taylor *et al*. (1993) |
| *Pseudomonas putida* KT2440 | Wild-type strain, derived from P. putida mt-2 (Worsey and Williams, 1975) cured of the TOL plasmid pWW0 | DSM 6125  Regenhardt *et al*. (2002) |
| GN442ΔPP_2426 | *P. putida* vanillin-accumulator strain  Δ*upp* ΔPP_0166-0168 Δ*vdh* ΔPP_3827-3832 ΔPP_2680 ΔPP_0545 ΔPP_1948 *lacIq*-*Ptac*-*ech-fcs* ΔPP_2426 | García-Hidalgo *et al.*, 2020 |
| TMB-JH001 | KT2440 carrying the plasmid pJH001 overexpressing *Cc-ATA* gene under IPTG induction, Sm^R^ | This study |
| TMB-JH002 | KT2440 carrying the plasmid pJH002 overexpressing *Cv-ATA* gene under IPTG induction, Sm^R^ | This study |
| TMB-JH003 | GN442ΔPP_2426 carrying the plasmid pJH001 overexpressing *Cc-ATA* gene under IPTG induction, Sm^R^ | This study |
| TMB-JH004 | GN442ΔPP_2426 carrying the plasmid pJH002 overexpressing *Cv-ATA* gene under IPTG induction, Sm^R^ | This study |
| TMB-JH006 | GN442ΔPP_2426 carrying the plasmid pJH004 overexpressing both *Cv-ATA* and *AlaDH* genes under IPTG induction, Sm^R^ | This study |
| TMB-NM011 | KT2440 carrying the plasmid pNM011 overexpressing *Pp-SpuC-II* gene under IPTG induction, Sm^R^ | This study |
| TMB-NM012 | KT2440 carrying the plasmid pNM012 overexpressing *Pp-Spuc-I*  gene under IPTG induction, Sm^R^ | This study |
| TMB-NM013 | KT2440 carrying the plasmid pNM013 overexpressing *Pp-ATA*  gene under IPTG induction, Sm^R^ | This study |
| TMB-NM014 | GN442ΔPP_2426 carrying the plasmid pNM011 overexpressing *Pp-SpuC-II* gene under IPTG induction, Sm^R^ | This study |
| TMB-NM015 | GN442ΔPP_2426 carrying the plasmid pNM012 overexpressing *Pp-SpuC-I* gene under IPTG induction, Sm^R^ | This study |
| TMB-NM016 | GN442ΔPP_2426 carrying the plasmid pNM013 overexpressing *Pp-ATA* gene under IPTG induction, Sm^R^ | This study |

*Antibiotic marker: Sm: Streptomycin.

**Table S4**. List of PCR primers utilized in this work

| **Primer name** | **Gene to amplify** | **Orientation** | **^1^Restriction enzyme 5’** | **^2^Primer sequence 5’🡪 3’** |
| --- | --- | --- | --- | --- |
| Fw_CC-TA | *Cc-ATA* | Forward | XbaI | TCG*TCTAGA***AGGAGGAAAAACAT**ATGGCAAACATTACAAACG |
| Rv_ CC-TA | *Cc-ATA* | Reverse | PsTI | AAAA*CTGCAG*TTATTGCTTTTGGGACTTCA |
| Fw_CV-TA | *Cv-ATA*/ | Forward | SacI | AAC*GAGCTC***AGGAGGAAAAACAT**ATGCAAAAACAAAGAACAAC |
| Rv_ CV-TA | *Cv-ATA* | Reverse | BamHI | CGC*GGATCC*TTATGCTAAACCTCTAGCCTT |
| Fw_PP-TA1 | *Pp-SpuC-II* | Forward | SacI | AAT*GAGCTC***AGGAGGAAAAACAT**ATGAGCGTCAACAACCCG |
| Rv_PP-TA1 | *Pp-SpuC-II* | Reverse | XbaI | CCCC*TCTAGA*TTATTGAATCGCCTCAAGGG |
| Fw_PP-TA2 | *Pp-Spuc-I* | Forward | SacI | AAT*GAGCTC***AGGAGGAAAAACAT**ATGAGTGAACAGAATTCGCA |
| Fw_PP-TA2 | *Pp-Spuc-I* | Reverse | XbaI | CCC*TCTAGA*TTACCGAACAGCCTCATAGG |
| Fw_PP-TA3 | *Pp-ATA* | Forward | SacI | AAT*GAGCTC***AGGAGGAAAAACAT**ATGAACGCGCCTTTCGCC |
| Rv_PP-TA3 | *Pp-ATA* | Reverse | XbaI | ACA*TCTAGA*TTACAGCTTGCCGACCAGCC |

^1^In *italic* the recognizing sequence for the respective enzyme.

^2^In **bold** the RBS sequence utilized

**Sequence S1**. Nucleotide sequence of the synthetic *AlaDH* gene from *B. subtilis* 168 (Genbank ID: 936557, Swiss-Prot: Q08352). The sites for the restriction enzymes *PsTI* (5`) and *SpeI* (3`) to clone *AlaDH* gene into the vector pSEVA424 are shown in red and the ribosome binding site (RBS) in italic. The start codon and the stop codon of the open reading frame (ORF) from *AlaDH* gene are shown in bold.

**CTGCAG***AGGAGGAAAAACCC***ATG**ATCATCGGTGTCCCAAAAGAGATTAAGAACAACGAAAACAGAGTTGCTTTGACTCCAGGTGGTGTTTCTCAATTGATTTCTAACGGTCACAGAGTTTTGGTTGAAACTGGTGCTGGTTTAGGTTCTGGTTTTGAAAATGAAGCTTACGAATCTGCTGGTGCCGAAATTATTGCTGATCCAAAACAAGTTTGGGATGCCGAAATGGTTATGAAGGTAAAAGAACCATTGCCAGAGGAATACGTCTACTTCAGAAAAGGTTTGGTCTTGTTCACCTACTTGCATTTGGCTGCTGAACCAGAATTGGCTCAAGCTTTGAAAGATAAGGGTGTTACTGCTATTGCTTACGAAACTGTTTCAGAAGGTAGAACCTTGCCATTATTGACTCCAATGTCTGAAGTTGCTGGTAGAATGGCTGCTCAAATTGGTGCTCAATTTTTGGAAAAACCCAAAGGTGGTAAGGGTATTTTGTTGGCTGGTGTTCCAGGTGTTTCTAGAGGTAAGGTTACTATTATTGGTGGTGGTGTTGTAGGTACTAATGCTGCTAAAATGGCTGTTGGTTTGGGTGCTGATGTTACCATTATAGATTTGAACGCCGACAGATTGAGACAATTGGATGATATTTTCGGTCACCAGATCAAGACGTTGATCTCTAATCCAGTTAACATTGCTGATGCTGTTGCTGAAGCTGATTTGTTGATTTGCGCTGTTTTAATTCCAGGTGCTAAAGCTCCAACTTTGGTTACTGAAGAAATGGTCAAACAAATGAAGCCAGGTTCCGTTATAGTTGATGTTGCTATAGATCAAGGTGGTATCGTTGAAACCGTTGATCATATTACTACCCATGATCAACCCACTTACGAAAAACATGGTGTTGTTCATTACGCTGTTGCTAATATGCCAGGTGCTGTTCCAAGAACTTCTACTATTGCATTGACTAACGTTACTGTTCCATACGCCTTGCAAATTGCTAACAAAGGTGCAGTTAAGGCTTTGGCTGATAATACTGCTTTGAGAGCTGGTTTGAATACCGCTAATGGTCATGTTACTTATGAAGCTGTTGCAAGAGATTTGGGTTACGAATATGTTCCAGCTGAAAAAGCCTTGCAAGATGAATCTTCTGTTGCTGGTGCT**TGAACTAGT**

**References**

García-Hidalgo, J., Brink, D.P., Ravi, K., Paul, C.J., Lidénb, G., and Gorwa-Grauslund, M.F. (2020) Vanillin Production in *Pseudomonas*: Whole-genome sequencing of *Pseudomonas* sp. strain 9.1 and reannotation of *Pseudomonas putida* *calA* as a vanillin reductase. *Appl Environ Microbiol* **86**:.

Graf, N. and Altenbuchner, J. (2014) Genetic engineering of *Pseudomonas putida* KT2440 for rapid and high-yield production of vanillin from ferulic acid. *Appl Microbiol Biotechnol* **98**: 137–149.

Regenhardt, D., Heuer, H., Heim, S., Fernandez, D.U., Strömpl, C., Moore, E.R.B., and Timmis, K.N. (2002) Pedigree and taxonomic credentials of *Pseudomonas putida* strain KT2440. *Environ Microbiol* **4**: 912–915.

Taylor, R.G., Walker, D.C., and Mclnnes, R.R. (1993) E.coli host strains significantly affect the quality of small scale plasmid DNA preparations used for sequencing. *Nucleic Acids Res* **21**: 1677–1678.

Worsey, M.J. and Williams, A.P. (1975) Metablism of toluene and xylenes by *Pseudomonas putida* (*arvilla*) mt 2: evidence for a new function of the TOL plasmid. *J Bacteriol* **124**: 7–13.
